# Age-dependent Phylodynamics with Application to Single-cell Lineage Trees

**DOI:** 10.1093/molbev/msag080

**Published:** 2026-03-25

**Authors:** Nicola Mulberry, Julia Pilarski, Jana Dinger, Tanja Stadler

**Affiliations:** Department of Biosystems Science and Engineering, ETH Zürich, Basel, Switzerland; Swiss Institute of Bioinformatics, Lausanne, Switzerland; Department of Biosystems Science and Engineering, ETH Zürich, Basel, Switzerland; Swiss Institute of Bioinformatics, Lausanne, Switzerland; Department of Biosystems Science and Engineering, ETH Zürich, Basel, Switzerland; Department of Biosystems Science and Engineering, ETH Zürich, Basel, Switzerland; Swiss Institute of Bioinformatics, Lausanne, Switzerland

## Abstract

As novel technologies for single-cell lineage tracing emerge, phylogenetic and phylodynamic tools are increasingly being used to study developmental processes. However, traditional phylodynamic methods rely on assumptions that are difficult to justify in developmental contexts. We present a generalization of the birth–death phylodynamic model to an age-dependent phylodynamic model. This method captures a key feature of development: due to cells dividing after characteristic generation times rather than after exponential waiting times, empirical cell lineage trees deviate from phylogenies generated under a birth-death model. By applying our method to a public dataset of stem cell colonies, we show how previous estimates of the underlying population-dynamic parameters were biased by the choice of a birth-death tree prior. We additionally showcase our method on embryonic lineage trees of an arthropod limb, demonstrating that age-dependence appears to be a common feature of development. Beyond developmental biology, our framework provides an approach for analyzing systems where classical birth-death assumptions may be violated or where empirical tree shapes are poorly captured by those expected under standard phylodynamic models. Our method is available as a BEAST2 package.

## Introduction

Modern tools in molecular biology are producing large-scale datasets that enable researchers to gain quantitative insights into developmental processes. Using single-cell RNA sequencing techniques, genetic material can be recovered at the resolution of individual genomes and on the scale of up to millions of cells per experiment. These sequencing methods are destructive, so that in most cases we cannot directly observe the cell population dynamics. A key challenge is thus to infer the temporal dynamics of a tissue or other cell population from molecular data obtained from a final sample of cells (Bunne et al. 2024; Askary et al. 2025). The complexity and scale of these datasets demands advancements in computational and statistical methods.

The potential for phylogenetic-based analyses in developmental biology has recently been highlighted (Wagner and Klein 2020; Stadler et al. 2021; Askary et al. 2025). A cellular phylogeny is a graphical representation of the lineage relationships between a sample of cells, where each tip represents a single sampled cell, and branches represent ancestral relationships. Such phylogenies can be inferred using either imaging techniques or genetic data (McKenna and Gagnon 2019). In the latter case, researchers could use naturally arising genetic variation, or sophisticated lineage tracing techniques to engineer genetic constructs with increased phylogenetic information content (Chan et al. 2019; McKenna and Gagnon 2019; Choi et al. 2022; Loveless et al. 2025). Once a phylogeny has been constructed, it serves as a statistical framework on which to analyze experimental data, such as transcriptomic measurements of cell states (Forrow and Schiebinger 2021).

Many phylodynamic methods, commonly employed in epidemiology and macroevolution, are based on the birth-death-sampling model (Gernhard 2008; MacPherson et al. 2022), in which a phylogeny is used to fit an underlying stochastic demographic process, together with a specified sampling process. In its basic form, this model assumes that the phylogeny was generated by a constant-rate birth-death branching process (Kendall 1948; Maddison et al. 2007), along with uniform sampling over extant lineages (Stadler 2010). These models make the convenient assumption that birth (or branching) events in a phylogeny occur according to a Poisson process, which assumes that the lifetimes of individuals being modeled are exponentially distributed, i.e. the process is Markovian. Modern methods have focused on adding complexity to these models by, for example, allowing birth and death rates to vary through time (the birth-death skyline) (Stadler et al. 2013), by adding population structure (Kühnert et al. 2016), or by incorporating complementary data sources (Kühnert et al. 2014; Andréoletti et al. 2022; Wright et al. 2022). Importantly, in these extensions, the process remains memoryless.

Previous work has noted the limitations of assuming exponentially distributed “lifetimes” across different fields. Evolutionary biologists have long-since debated the possibility of age-dependence in speciation and extinction rates (Morlon 2014). To this end, phylodynamic methods have been developed to estimate age-dependent extinction rates, based on both fossil records and extant phylogenies (Alexander et al. 2016; Hagen et al. 2018). In epidemic modeling, it is common practice to add either a latent period or multiple infectious stages to a compartmental model in order to capture non-exponential dynamics. Such compartmental models can been incorporated into phylodynamic analyses under approximate (coalescent) (Volz and Siveroni 2018) or exact (King et al. 2025; Vaughan and Stadler 2025) frameworks. These methods tend to be computationally intensive and limited in the number of compartments one can model. In this work, we take a distinct approach, and develop a method with general age-dependence (not limited to a small, pre-specified number of compartments).

There are several challenges in applying phylogenetic and phylodynamic tools to single-cell genomic data. First, to reconstruct cell phylogenies from sequencing data, we need to accurately describe the mutation process. This is particularly important when working with lineage tracing data, where standard assumptions about molecular evolution are violated. This problem has been addressed in previous research (for example, the BEAST2 packages developed in Seidel and Stadler 2022; Seidel et al. 2024; Zwaans et al. 2025). Second, to quantify cell population dynamics from phylogenies, we need to accurately model the underlying tree-generating process. While age-dependent branching processes have long-since been used to model cellular dynamics (Kimmel and Axelrod 2015), their incorporation into phylogenetic analysis has been lacking.

We present a phylodynamic method which generalizes the traditional birth-death approach. Specifically, we introduce non-Markovian dynamics: each lineage’s branching rate depends on its internal *age*, and not on an external time scale. Age-dependent processes are well studied in the branching process literature, however, they have seen less application in the field of phylodynamics (Lambert and Stadler 2013; Morlon 2014; Alexander et al. 2016). While the likelihood presented here was first derived in Jones (2011a), this is, to our knowledge, the first implementation and application of the method. We furthermore present an efficient and scalable approximation to the exact likelihood which allows us to perform inference in a Bayesian setting. We implement the likelihood calculation in BEAST2 (Bouckaert et al. 2019), enabling Bayesian phylodynamic analysis under the age-dependent branching (ADB) model. As a proof-of-concept, we apply our package to lineage recordings of mouse embryonic stem cells (Chow et al. 2021). As a result, we characterize the cell population-dynamic parameters and find that parameters inferred from incomplete cell phylogenies under age-dependent branching strongly agree with estimates obtained from ground-truth cell population trees, while the estimates differ when using the traditional birth-death model. Finally, we apply our method to the cell lineage of a developing arthropod limb (Wolff et al. 2018), and find support for age-dependent dynamics in organ development.

## Model

**Figure 1:**
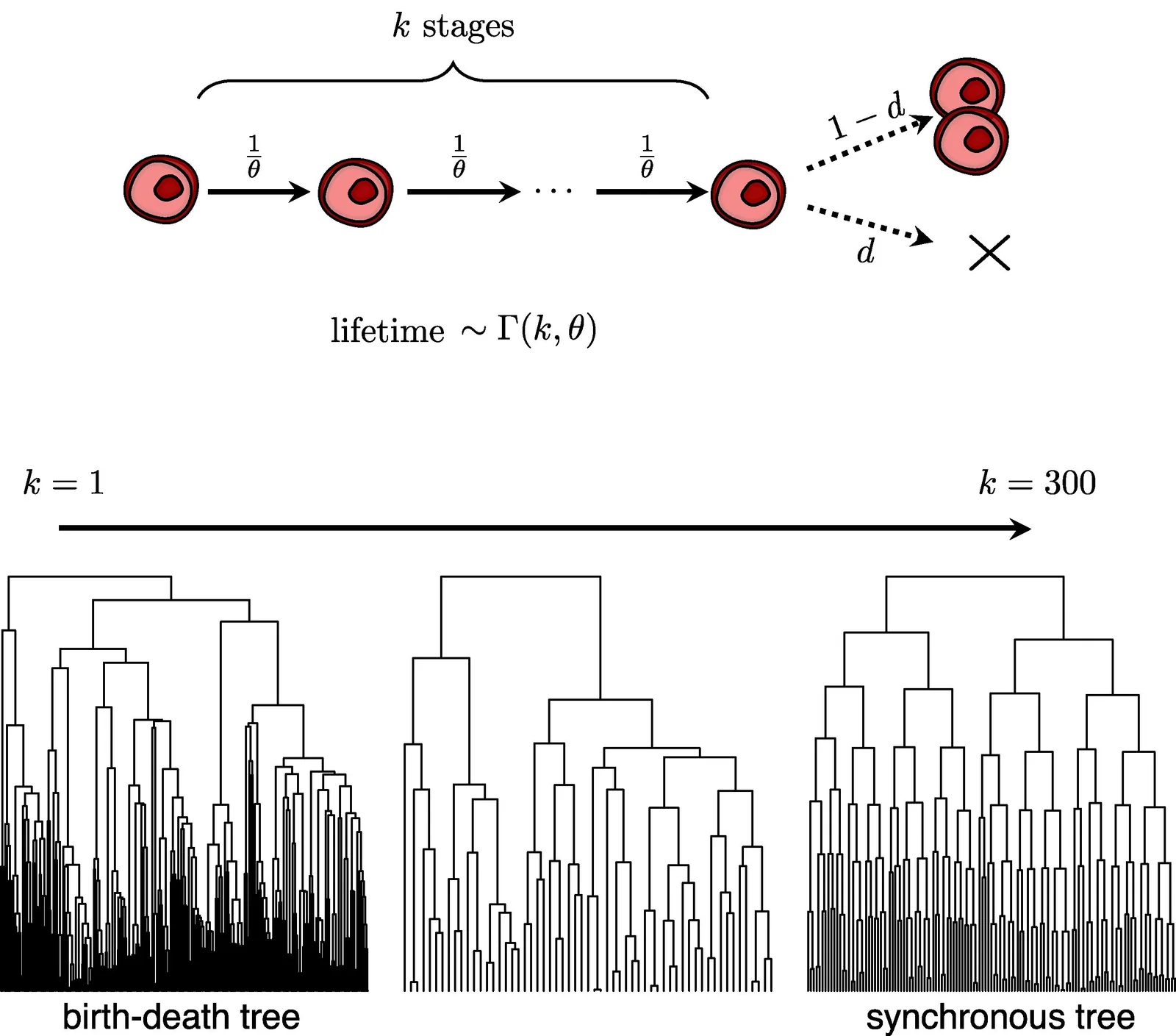
(Top) Our method is based on an age-dependent branching process, where we additionally make the assumption that cell lifetimes are Erlang distributed with shape parameter *k* and scale parameter *θ*. At the end of its lifetime, the cell either dies with probability *d*, or divides into two new cells with probability (1−d). Solid arrows denote rates, dashed arrows denote probabilities. The birth-death model is a special case of our model found by setting k=1, with corresponding birth rate λ=(1−d)/θ and death rate μ=d/θ. (Bottom) Examples of different tree shapes under various shape parameters *k*. As *k* increases, the trees become more and more synchronous.

In our underlying population-dynamic model, we assume that cells live for some amount of time, after which they either die with probability *d* or otherwise divide, leaving behind two daughter cells. Crucially, the rate at which a cell terminates – resulting in either two daughter cells or a death – increases with age. Upon division, the age of each daughter cell is set to 0, and the process repeats. This is a significant departure from the birth-death model, wherein division and death occur at some specified rate (either constant in time or dependent on an external time scale). At some final time, extant cells are sampled with uniform probability *ρ*; the resulting phylogeny displays the ancestral relationships among this sample of cells. Here, we model cell lifetimes as being Erlang distributed, and parameterize our model by an integer shape parameter k∈N and mean lifetime $\ell \in R_{+}$. This distribution is highly flexible, and has additionally been shown to be a good fit to empirical cell lifetimes (Yates et al. 2017). One could interpret the shape parameter *k* as, for example, stages or sub-stages in a cell cycle. This model and its effect on tree shapes is illustrated in Fig. 1.

Section 5.1 presents the derivation for the likelihood of a phylogeny under this model. Note that a lineage is included in the phylogeny only if it has at least one sampled ancestor at the present. While a more detailed derivation is given in Jones (2011a), we present a condensed version for the single-type case here. Unlike in the Markovian birth-death case, this likelihood has no analytical form, and is instead described by a system of integral equations. In our efficient implementation of the likelihood, we eliminate the need to solve an integral equation over each branch in the tree. Details on our numerical methods as well as our approximations to the exact likelihood are given in Section 5.2.

## Results

### Implementation & validation

We first verify the correctness of our implementation of the ADB likelihood by comparing to the analytical solution for k=1 (SI Section C). While we generally see good agreement between the methods, we nonetheless observe significant errors under low sampling proportion (Figs. S3 and S4). These errors are numerical, and not a result of our approximation: they are present in our exact implementation of the likelihood, and can be controlled by decreasing the step size of the method (Fig. S4). In particular, we require a sampling proportion of ρ≳0.01 to achieve sufficient accuracy when k=1. However, this discrepancy appears to decrease as the shape parameter *k* increases (Fig. S3).

**Figure 2:**
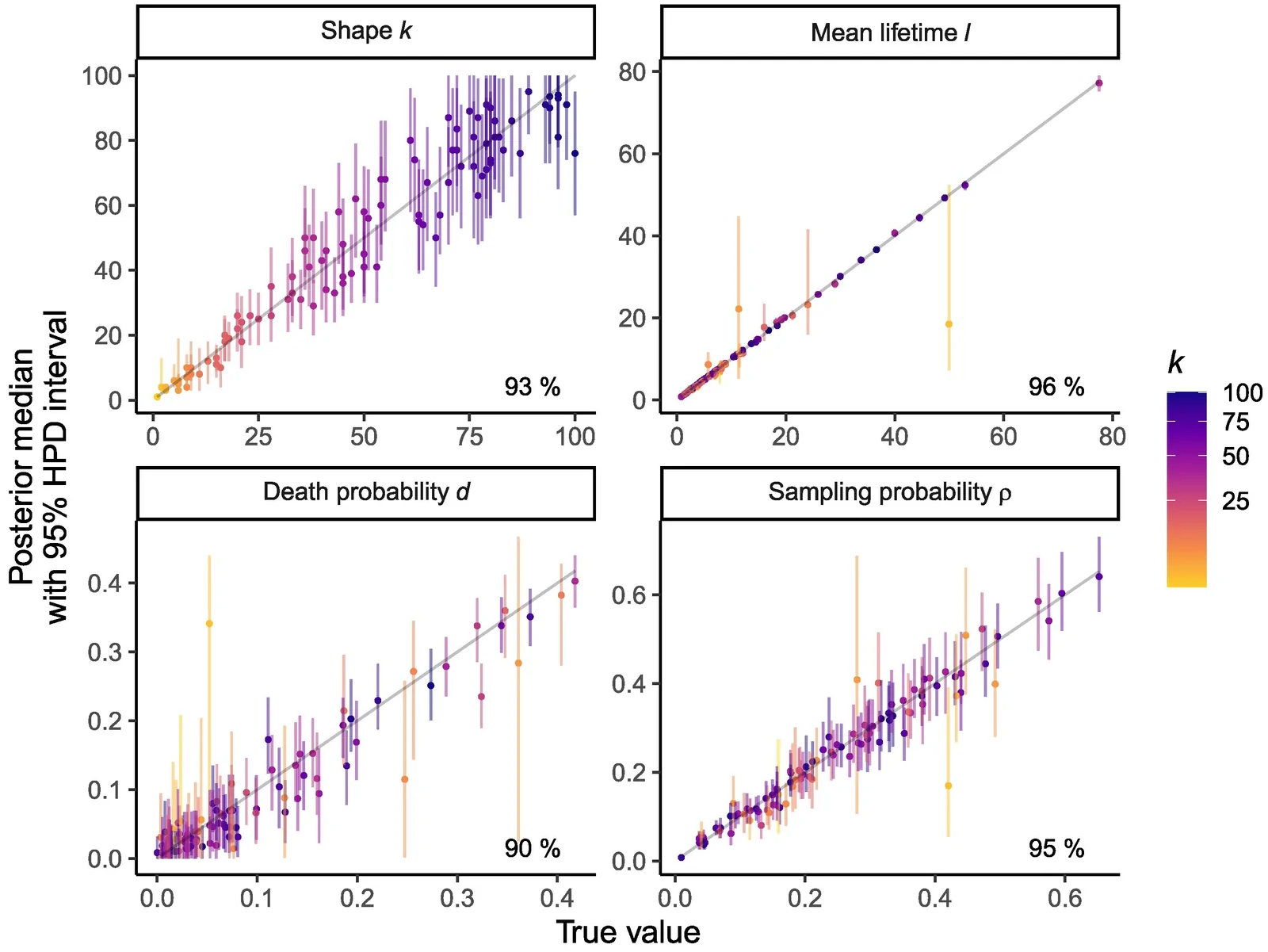
Validation of ADB model on simulations. Panels show the true parameter values (*x*-axis) plotted against the posterior estimates (*y*-axis) for 99 out of 100 simulations for which the MCMC run reached convergence. Dots indicate the medians, bars the 95% HPD intervals, and the diagonal line shows x=y. Simulations are colored by their true shape parameter *k*. The percentage summarizes the coverage (considering all 100 simulations).

Applying Bayesian phylodynamic inference to simulated phylogenies shows that our method estimates all ADB model parameters—shape *k*, mean lifetime ℓ, death probability *d*, and sampling probability *ρ*—reliably. The inferred parameters are well-correlated with the true values and satisfy the 95% coverage criterion (Mendes et al. 2025) at a tree size of 100. (Fig. 2). Further details are provided in Sections 5.5 and 5.6. In general, mean lifetimes are inferred most accurately, while death probabilities exhibit the largest bias. This bias becomes more pronounced on larger trees, but does not hinder the inference of the remaining parameters (cf. simulation study on trees with 1000 tips in the SI).

It is well known that for the constant-rate birth-death-sampling model, only two of the three parameters {λ,μ,ρ} are identifiable (Stadler 2009). Typically, researchers will fix either the death or the sampling rate and infer the remaining two parameters under the fixed rate. The simulation study shown in Fig. 2 shows how we recover this non-identifiability as the shape parameter *k* becomes small. However, we observe improved accuracy and precision of *k*, ℓ and *ρ* estimates with increased shape parameter, for which the branching patterns become more regular. Hence, we hypothesize that the model becomes identifiable for large *k*. In practice, we observe that all four parameters tend to be well-recovered when k≳20 (see SI for more details).

We next perform an additional simulation study to investigate the low sampling regimes (ρ<0.02, Fig. S11). Briefly, numerical errors accumulate when k≲20 and analyses tend to not converge. In the case of higher *k*, however, the analyses converge and the true parameters are recovered in over 75% of simulations (see SI for more details).

**Figure 3:**
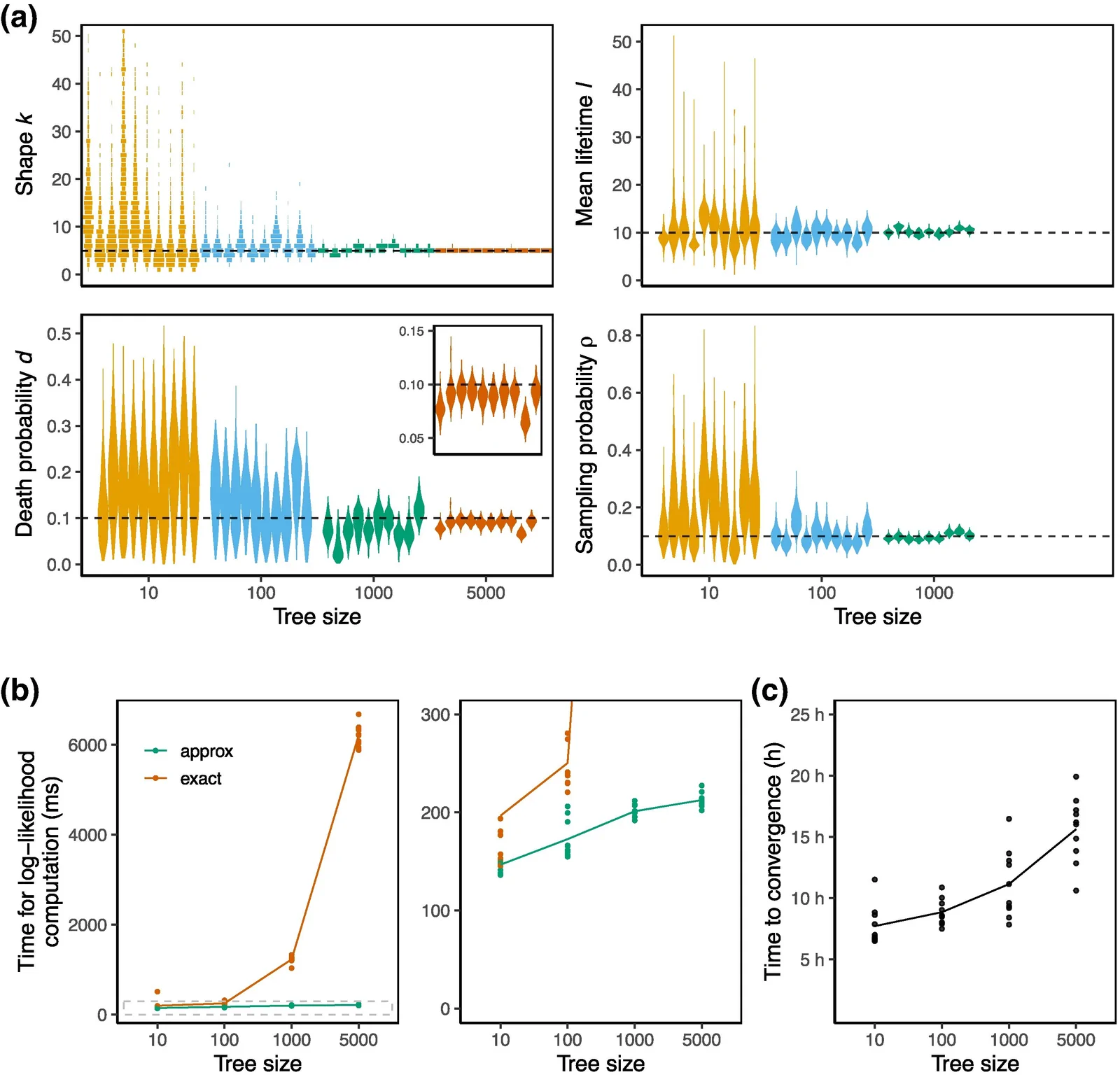
Phylodynamic inference under ADB model on simulated trees of varying size. a) Posterior distributions of inferred parameters per simulation, grouped by tree size (i.e. number of tips). Dashed lines indicate true parameter values. The inset highlights a small systematic bias in the estimated death probabilities for large trees. b) Runtime of phylodynamic log-likelihood computation with exact or approximate edge densities. Dots represents individual trees, lines connect means per tree size. The dashed area in the left panel is magnified on the right. c) Inference runtime until MCMC reached convergence threshold (posterior ESS >200). Each dot represents an MCMC chain for a simulated tree.

Further, we simulate phylogenetic trees of varying sizes, ranging from 10 to 5000 tips, under a fixed set of parameters. Overall, the posterior distributions of parameters inferred from larger trees are narrower and more tightly centred around the true values (Fig. 3a), as expected with an increase in data. Again, we observe a small systematic bias in the inference of death probabilities, which becomes more apparent on larger trees. We hypothesize that the bias results from errors in approximating the branch densities (cf. Section 5.2 and SI). This bias is a trade-off for massively reducing the computational cost of the phylodynamic likelihood calculation. For trees with 5000 tips, a single calculation of the full likelihood (which solves an integral equation over each branch in the tree) takes roughly 6s (Fig. 3b), meaning an MCMC chain with 1M steps would run for ≈70 days. By contrast, our approximation leads to convergence, i.e. reaching an effective sample size (ESS) of at least 200, in 10–20h (Fig. 3c).

### Application

#### Mouse embryonic stem cells

We demonstrate the ADB model on the experimental *in vitro* dataset of intMEMOIR recordings in mouse embryonic stem cells (Chow et al. 2021), as available during the DREAM challenge (Gong et al. 2021). In the experiment, cells growing into colonies were traced using genetic lineage barcoding and time-lapse microscopy. Crucially, ground-truth cell phylogenies and cell population trees per colony have been extracted from the recordings. These provide us the rare opportunity to evaluate how well a tree-generating model captures the dynamics of the empirical system.

**Figure 4:**
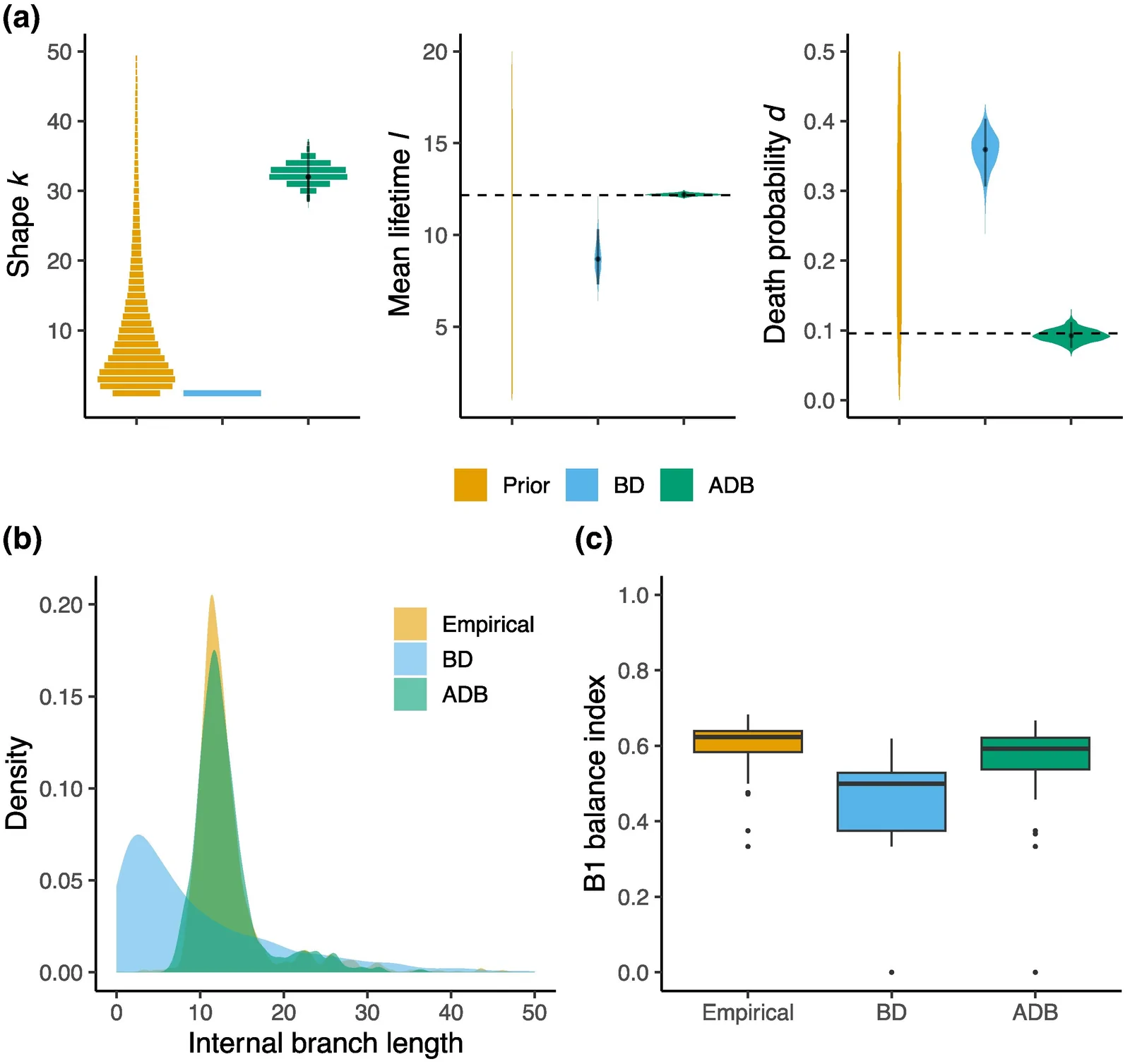
Application of phylodynamic models to single-cell phylogenies from mouse embryonic stem cell colonies. a) Prior and posterior distributions of phylodynamic parameters inferred from cell phylogenies under the BD and ADB models. Dashed lines indicate the mean lifetime $\hat{\ell }$ and death probability $\hat{d}$ calculated from ground-truth cell population trees (obtained from time-lapse microscopy) for reference. b) Internal branch length distribution, and c) B1 balance index of empirical (ground-truth) cell phylogenies from the experiment and simulated cell phylogenies.

We quantify the population dynamics of stem cells using Bayesian phylodynamic inference (Fig. 4a). Under the ADB model, we infer the shape parameter *k* at a posterior median of 32 (95% HPD interval: [30,35]). This estimate differs substantially from k=1, indicating a higher level of synchronicity and regularity of cell divisions in the population than would be expected under the birth-death (BD) model. Further, we estimate the mean lifetime ℓ of cells to be 12.20h ([12.06,12.32]) and the death probability *d* to be 0.092 ([0.075,0.112]). These ADB estimates do not overlap with BD estimates, highlighting that phylodynamic parameters are closely linked to tree shape. Moreover, the posterior distributions of ℓ and *d* are narrower under the ADB model compared to the BD model, indicating a higher level of confidence in our estimates. Note that we here fix the sampling proportion *ρ* to the true value, as was done in Seidel and Stadler (2022).

The inferred parameters are based on phylogenies which delineate the lineage history of only a sample of cells and thus lose information about direct parent-offspring relationships and timings of cell divisions and deaths (Stadler et al. 2021). To assess whether our proposed phylodynamic model quantifies the cell population process correctly based on the incomplete cell phylogenies, we derive the mean lifetime $\hat{\ell }\approx 12.17$ and death probability $\hat{d}\approx 0.096$ directly from the complete cell population trees. Notably, the empirical estimates fall in the 95% HPD intervals of parameters inferred under the ADB model, but not the BD model. Finally, we compare the empirical cell phylogenies to phylogenies simulated under the inferred BD and ADB models. We observe a strong agreement of the internal branch length distribution and tree balance between empirical phylogenies, and phylogenies simulated under the ADB model (Fig. 4b/c).

#### Arthropod limb

**Figure 5:**
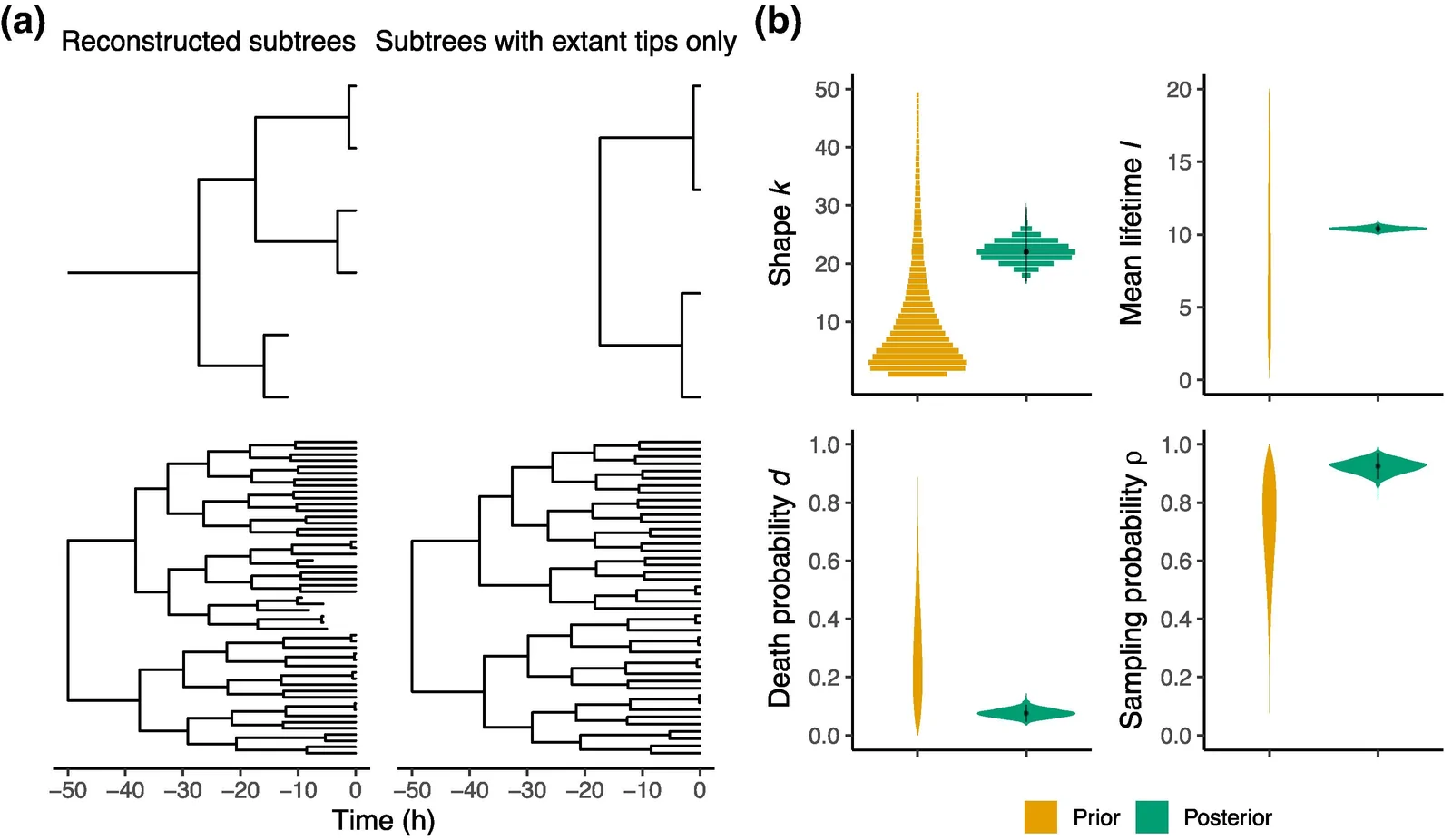
Application of ADB to the cell lineage of an arthropod limb. a) Input data. Shown are two example subtrees extracted from the *Parhyale* T2 limb cell lineage, as reconstructed from light-sheet imaging (left) and as processed for inference (right). b) Prior and posterior distributions of phylodynamic parameters inferred from phylogenies (subtrees with extant tips only) under the ADB model.

Next, we apply our package to quantify *in vivo* limb development of the crustacean *Parhyale hawaiensis*. Wolff et al. imaged *Parhyale* embryogenesis using multi-view light-sheet microscopy, and reconstructed the lineage of an outgrowing thoracic limb at single-cell resolution using the MaMut software (Wolff et al. 2018). Salvador-Martínez et al. published the lineage in a convenient format on the platform CeLaVi (Salvador-Martínez et al. 2021). Here, we extract subtrees from the lineage with extant cells only (cf. Fig. 5a), and infer phylodynamic parameters under the ADB model.

The posterior distributions for each inferred parameter are shown on Fig. 5b. We estimate the shape parameter *k* to be 22 ([18,25], $\hat{R}=1.0013$), and infer a mean lifetime ℓ of 10.41h ([10.12,10.68], $\hat{R}=1.0050$), death probability *d* of 0.076 ([0.047,0.105], $\hat{R}=0.9987$), and sampling probability *ρ* of 0.93 ([0.88,0.97], $\hat{R}=1.0003$). The estimated mean lifetime falls within the range reported in the original study (cell cycle length of ≈ 7–16h) (Wolff et al. 2018). Notably, we see no posterior support for birth-death dynamics (k=1). Further details on this analysis are given in Section 5.7.

## Discussion

It is typically not possible to directly observe the full dynamics giving rise to a population of cells; this is particularly true if our only data comes from endpoint single-cell sequencing. However, phylogenetic and phylodynamic tools promise to shed light on these hidden processes. That said, the application of these tools to the developmental context poses numerous challenges on both the phylogenetic and phylodynamic levels. Here, we propose a method to overcome a key challenge on the phylodynamic level: we replace the commonly used Markovian birth-death model with an age-dependent model, taking into account that cell divisions are typically more synchronized than modeled by the traditional birth-death process.

Indeed, our analysis of a single-cell lineage tracing dataset where ground-truth trees are available highlights that estimates with our novel approach agree with the ground-truth while the classic birth-death approach leads to biased estimates. Furthermore, our results on the arthropod limb indicate that coordinated cell behaviors, giving rise to tissues and organs, show evidence for age-dependence. This analysis also showcases that population-dynamic parameters can be jointly quantified based on fragmented cell lineage trees from a common developmental process. Despite improvements in cell tracking, lineages reconstructed from light-sheet microscopy typically contain missing links or truncated branches. Phylodynamic modeling enables estimating the probabilities of cell divisions, deaths and dropouts from such incomplete data.

Our model assumes that all cells are homogeneous, while typical cell populations are heterogeneous, containing cells in different developmental stages. A straightforward extension of our method would be to incorporate multiple cell types, each with its own lifetime and death parameters. We could model the type changes that occur as cells differentiate, which affects the growth dynamics of the population, and hence, the tree shape. In this way, phylogenies may provide additional insights into differentiation trajectories. This method would be an extension of the birth-death multi-type (BDMM) method (Kühnert et al. 2016; Vaughan and Stadler 2025). While our current model is suitable for studying systems such as stem cells growing in culture, a multi-type version of the model would be suitable for studying more complex cell populations and tissues developing in vivo.

The current version of the ADB package suffers from a number of computational limitations. While our implementation enables us to infer phylodynamic parameters in a Bayesian setting on trees of size up to ≈5000 tips, it nonetheless comes with significant computational costs over the standard, single-type birth-death model (as expected, since the birth-death model has an analytical expression for the likelihood). It is therefore recommended to only use the package when there is prior expectation of age-dependence. We additionally suffer from significant numerical errors in certain parameter regimes, most notably for very low sampling proportions (less than approx. 1%). This currently poses a problem for applying the method to many developmental data sets which are sparsely sampled. Such regimes may warrant different approaches and approximations, which are left for future work. In addition, our approximation for the edge densities is less accurate under low death probabilities, however we are still able to recover the true parameters in this regime, up to a slight bias in the death parameter on large trees.

We have currently implemented and validated this model as a phylodynamic likelihood; that is, given a fixed phylogeny, we can connect the tree to the underlying population dynamic model. The phylogeny itself is assumed to be either known or inferred from, for example, single-cell lineage tracing data (Feng et al. 2021; Seidel and Stadler 2022; Seidel et al. 2024; Zwaans et al. 2025). One can also use the phylodynamic model as a tree prior in a full joint Bayesian inference (Bouckaert et al. 2019). However, in our case, joint inference poses additional difficulties since this is a broader tree prior, and the relationships between the population-dynamic parameters and tree space has proved challenging to deal with. We hypothesize that new, non-trivial, performant operators are required and leave this for future work.

Identifiability in the context of birth-death phylodynamics is an important concern (Louca and Pennell 2020), even when restricting to a class of constant-rate models (Stadler 2009). In brief, in the constant-rate birth-death case, we can only find two out of the three birth, death, or sampling rates. It is therefore important to investigate identifiability issues under our model, first within the class of constant-rate models. Our initial results, both from simulation studies and from investigating the lineage-through-time plots, indicate that as the true underlying shape parameter increases, we can recover all four population-dynamic parameters (those being, in our case, the shape, lifetime, death and sampling parameters). For shape parameters closer to one, we recover the known constant-rate non-identifiability, at least in the practical sense. A proof for this (non-)identifiability is, however, currently lacking, and will be left for future work.

Noting the computational and theoretical limitations discussed above, we can make practical recommendations for the usage of the ADB package. If k≲20, we are limited to analysing lineage trees with a sampling proportion of at least approx. 1%. In this regime, we also suggest putting informative priors on either the death probability *d* or the sampling proportion *ρ* due to non-identifiability concerns. However, if k≳20, we are able to estimate all model parameters; in this case, we can also perform inference in lower sampling regimes. We note that in both of our empirical analyses, we tend to infer shape parameters which meet this criterion. Further, the analyses are prone to numerical errors and convergence issues when the death probability *d* is greater than 0.4; these problems increase as *d* increases further (although, we note that a parameter regime where the probability of cell death, compared to division, being above 40% is typically not biologically relevant). Finally, we reiterate that, as a result of our approximation, we tend to see a slight underestimation of the death probability across all regimes, especially on large trees.

While the motivation behind this method comes from developmental biology, our method would be applicable to any phylodynamic setting where age-dependence could be a factor, and where birth-death trees do not adequately describe empirical tree shapes. For example, certain species trees have been shown to be less balanced than expected under the birth-death-sampling model (Jones 2011b; Hagen et al. 2015), a phenomenon which could be captured in our model by taking a shape parameter *less* than one. This would be incompatible with our current approximation, however alternative approaches to address scalability could rely instead on the high sampling rates common in such settings (Maliet and Morlon 2022).

Eventually, we envision that ADB will be fully integrated into the pipeline for Bayesian phylogenetic analysis of single-cell data. For analyzing single cell sequences, users would be able to leverage recent single-cell-related developments in the BEAST2 ecosystem, including packages tailored to specific editing models (Seidel and Stadler 2022; Seidel et al. 2024), as well as explicit error models (Chen et al. 2022). In this pipeline, ADB would serve as a biologically-motivated and informed tree prior. Together, such analyses will enable joint inference of both the underlying phylogeny and population dynamics directly from sequence data in a way that is statistically robust and grounded in single-cell biology. With the increasing amount of single cell lineage tracing technologies and data becoming available, this computational framework has the potential to lead to exciting novel biological discoveries.

## Methods

### Governing equations

We describe a binary branching process in which the lifetime of a cell is distributed according to a distribution with probability density function f(a) and cumulative density function F(a), where *a* denotes the age of the cell. At the end of a cell’s lifetime, it dies with probability *d*, else it divides and generates two daughter cells. We will assume that d<1/2. After a fixed amount of time, the extant cells are sampled uniformly with probability *ρ*. Following convention, we track time backwards from the present, with t=0 denoting the present day/sample time and $t=t_{or}$ being the origin time of the process.

We will first derive $P_{0}(t)$, the probability that a cell originating at time *t* will have no descendants in the sample. We will then use this quantity to derive $P_{1}(t)$, the probability that a cell born at time *t* leaves exactly one sampled descendant, and also b(s,t), the probability that a cell which originated at time *t* had exactly one descendant at time *s*. In this process, there is a fundamental difference between $P_{1}(t)$ and b(s,t): at the tips a cell’s lifetime is censored due to sampling, whereas along an edge, we observe at least one complete life-cycle.

#### No sampled descendants

Consider a cell which originated at time *t*, where time is counted backwards from the present (t≡0). There are three possibilities for the cell to have no descendants at present day: either the cell persists for time *t* and then is not sampled at present, it terminates sometime before the present and leads to a death, or the cell divides after some time *w* but its children have no descendants. Therefore, we have


(1) $$\begin{array}{r} P_{0}(t)=(1-\rho )\left(1-F(t)\right)+dF(t)+(1-d)\int_{\;0}^{t}\;f(t-w)P_{0}{\left(w\right)}^{2}\;dw. \end{array}$$

#### Single sampled descendant

A cell originating at time *t* can have exactly one sampled descendant if it survives until present and then is sampled, or if it divides at some time w∈(0,t) leading to two daughter cells, one with no sampled descendants and the other with exactly one (but it does not matter which). We get


(2) $$P_{1}(t)=\rho (1-F(t))+2(1-d)\int_{\;0}^{t}\;f(t-w)P_{0}(w)P_{1}(w)\;dw.$$

#### Density of an edge (s,t)

Consider a cell which originated at time *t*. We wish to work out the probability that this cell has exactly one descendant at time s>0. To compute this quantity, we consider again two possibilities. Either the original cell survives for time t−s and then divides, or there is a division at some time w∈(s,t) which results in two daughter cells. As before, one daughter cell must leave no sampled descendants. The other daughter cell must result in the edge now between time *s* and *w*. This gives


(3) $$b(s,t)=(1-d)f(t-s)+2(1-d)\int_{\;s}^{t}f(t-w)P_{0}(w)b(s,w)\;dw.$$

#### Phylodynamic likelihood

Consider a reconstructed tree $T_{r}$ on *n* tips. Let t=0 denote the present day, and $t=t_{or}$ be the time-of-origin of our process. Let E denote the external edges of $T_{r}$ andI denote the internal edges of $T_{r}$, and let $\left(s_{e},t_{e}\right)$ denote the start and terminus of edge *e*. The densityof a tree is then


(4) $$P(T_{r}\;|\;t_{or})=\frac{1}{1-P_{0}(t_{or})}\prod_{e\in E}P_{1}(t_{e})\prod_{e\in I}b(s_{e},t_{e}).$$

This is essentially the likelihood given in Jones (2011a), except here we condition on the survival of the process. Considering the likelihood of a labelled tree instead of an unlabeled one, we multiply the equation by factor $2^{n-1}/n!$ (Gernhard 2008).

For example, in the case of a tree with no extinct or unsampled lineages (ρ=1 and d=0), we get


$$P(T_{r}\;|\;t_{or})\propto \prod_{e\in I}f(t_{e}-s_{e})\prod_{e\in E}(1-F(t_{e})).$$

This corresponds to probability of the observed lifetimes, as well as the censored lifetimes at the tips. It is readily verified that Equation (4) simplifies to the analytical expression for the birth-death model with extant sampling (Stadler 2010) when the lifetimes are exponentially distributed.

Alternatively, we can condition on the root of the reconstructed tree, that is, the time of the first cell division event (cf. Stadler 2010). Let $t=t_{root}$ and let $T_{r}^{L}$ and $T_{r}^{R}$ denote the left and right subtree of $T_{r}$ descending from the root. Then


(5) $$P(T_{r}\;|\;t_{root})=P(T_{r}^{L}\;|\;t_{or}=t_{root})P(T_{r}^{R}\;|\;t_{or}=t_{root}).$$

Again, we multiply the equation by factor $2^{n-1}/n!$.

### Computing the phylodynamic likelihood

Equations (1)–(3) can be solved using fixed point iterations (as suggested also in Jones 2011a). For example, to solve for $P_{0}(t)$ (1), we take $X_{0}=(1-\rho )(1-F(t))+dF(t)$ and iterate:


$$X_{i+1}=X_{0}+(1-d)\int_{\;0}^{t}f(t-w)X_{i}^{2}(w)\;dw,$$

until a convergence criterion is reached. The convolution term can be solved efficiently using the FFT. For the edge probabilities, we consider b(s,t) to be a function of *t* for each time *s*. Therefore, for each edge $e=(s_{e},t_{e})\in I$ , we solve $b(s_{e},t)$ for $t\in [s_{e},t_{e}]$, and then evaluate $b(s_{e},t_{e})$. While each equation can be solved relatively efficiently using the procedure outlined above, since we are required to solve an integral equation over each internal edge, this computation is not scalable to large trees. We instead present an approximate likelihood which is scalable and can be used in a Bayesian inference setting.

#### Approximating edge densities

We now let the cell lifetimes be Erlang distributed. That is, a∼Γ(k,θ) with integer shape parameter *k*. Under this assumption, we can approximate the edge densities by an explicit equation. Let ${\bar{P}}_{0}$ denote the average value of $P_{0}(\tau )$ over the edge. Then we let


(6) $$b(s,t)\approx \sum_{n=0}^{n_{u}}2^{n}(1-d{)}^{n+1}f(t-s;(n+1)k,\theta ){{\bar{P}}_{0}}^{n},$$

for some finite $n_{u}$ (see SI for derivation). The number of terms taken in the above approximation depends on a user-specified parameter ϵ.

We find that for k>1, solving Equation (6) is more efficient and scalable than solving the original integral equation for b(s,t) (3). We note that, under the Erlang assumption, we could instead recast the integral equations as systems of ordinary differential equations by tracking each transitional state explicitly. However, as this approach involves solving a system of *k* equations, it scales poorly with *k* and is not pursued here.

### Lineages through time

Consider a cell that originated at time *τ* in the past. Let μ(t,τ) be the expected number of lineages at time 0≤t≤τ descending from this cell. Following, e.g. Kimmel and Axelrod (2015), we get:


(7) $$\mu (t,\tau )=1-F(\tau -t)+2(1-d)\int_{\;t}^{\tau }f(\tau -w)\mu (t,w))\;dw.$$

To see this, we first consider that the original cell survives until time *t*, in which case the expected number of lineages stays the same. Otherwise, the cell divides at some time w∈(t,τ) with probability f(τ−w) and survives with probability (1−d), leaving two daughter lineages, each generating μ(t,w) expected lineages at time *t*. This includes all lineages even if they do not appear in the reconstructed phylogeny, and is conditioned on the time of origin of the process.

In the reconstructed phylogeny, we include a lineage only if it has at least one sampled ancestor at the present. Therefore, the expected number of lineages in $T_{r}$ becomes


(8) $$\begin{array}{rl} \mu (t,\tau ) & =(1-P_{0}(\tau ))(1-F(\tau -t))\\ & \;+2(1-d)\int_{\;t}^{\tau }f(\tau -w)\mu (t,w)\;dw. \end{array}$$

For example, the expected final size is given by $\mu (0,\tau_{or})$. Typically, we will also condition on the survival of the process.

### Tree simulation under ADB

We introduce the R package *scTreeSim* for simulating trees under the age-dependent branching model. In *scTreeSim*, phylogenetic trees with (1) a predefined number *n* of extant tips or (2) a predefined time of origin $t_{or}$ can be simulated. We start with a single cell and sample its lifetime from a Gamma distribution with shape k>0 and scale θ>0. At the end of its lifetime, the cell divides with probability (1−d) or dies with probability *d*. In the former case, we sample the lifetimes of its two daughter cells. We continue the process until (1) $\frac{n}{\rho }$ cells are alive at a time, where *ρ* is the sampling probability of extant cells, or (2) the defined time $t_{or}$ has passed. Next, we set the time to t=0, censor the lifetimes of all living cells, and thus obtain the complete tree. Next, we prune dead cells and sample extant cells uniformly with probability *ρ*. The tree is rooted, binary and ultrametric. In the SI, we validate the simulated trees against the expected LTT curves given by Equation (8).

Note that in (1) we simulate the process forward in time until the specified number of living cells is first reached. This procedure, referred to as the simple sampling approach (SSA) can produce biases in the tree origin and the length of external branches (Hartmann et al. 2010), as later periods with the same number of living cells are disregarded. In practice, however, these biases only become apparent for higher death probabilities, d≳0.4. In this study, we apply our method to lower *d* regimes, hence, we simulate trees with SSA. We provide the general sampling approach (GSA) (Hartmann et al. 2010; Stadler 2011) as an option in *scTreeSim* and recommend using it for higher *d*.

### Bayesian inference

Consider a time-scaled phylogenetic tree T obtained from empirical observation or computational reconstruction. The goal is to characterize the tree-generating process, that is, the population dynamics which resulted in the observed phylogeny. Using Bayes’ rule, the posterior distribution of phylodynamic parameters is given by


(9) P(k,ℓ,d,ρ|T,t)∝P(k)P(ℓ)P(d)P(ρ)P(T|k,ℓ,d,ρ,t).

Here, shape k≥1, mean lifetime ℓ>0, death probability d∈[0,1] and sampling probability ρ∈[0,1] parametrize the ADB process with Erlang-distributed lifetimes. The phylodynamic likelihood P(T|k,ℓ,d,ρ,t) is given by Equation (4). For inference, prior distributions can be specified for each parameter. In BEAST2, the posterior distribution is approximated by efficiently sampling from the parameter space with the Markov chain Monte Carlo (MCMC) algorithm.

### Simulation study

First, we simulate 100 phylogenetic trees with 100 tips under the ADB process using *scTreeSim*. For each simulation, we draw parameters from the prior distributions


$$\begin{array}{r} \begin{array}{cc} k∼\text{Uniform}(1,100) & \;[3,98]\\ \ell ∼\text{LogNormal}(2,1) & \;[0.19,38.33]\\ d∼\text{Exponential}(10) & \;[6.34\times 10^{-10},0.30]\\ \rho ∼\text{Beta}(2,5) & \;[0.02,0.59] \end{array} \end{array}$$

with 95% highest density intervals denoted in brackets. A uniform distribution on the shape parameter allows us to explore the wide range of tree shapes, from constant rate birth-death to more and more synchronous trees. We choose the other prior distributions to be biologically realistic, in particular having d<0.5. Here, we limit ρ≳0.01, but perform simulations in lower sampling regimes in the SI.

We then infer k,ℓ, *d* and *ρ* from the trees (with known time of origin) using the BEAST2 package ADB. For each tree, we run an MCMC chain of length 1M for 24h, or until effective sample size (ESS) is 200. We use a sampling frequency of 1,000 and discard a 10% burn-in. We assert convergence by computing the revised Gelman-Rubin (GR) statistic $\hat{R}$ (Vats and Knudson 2021) per chain and parameter, assuring that it approaches 1 and falls below the threshold (as advised in the documentation of the R package *stableGR*). For each parameter, we summarize the posterior distribution by calculating the median and 95% highest posterior density (HPD) interval.

Note that for one out of 100 trees, the inference did not reach convergence. In this case, the MCMC chain entered an erroneous parameter regime (*d* approaching 0.5 and ρ≲0.001, as discussed in SI, Section B), leading to numerical instability in the likelihood calculation and unreliable parameter estimation. These issues are clearly visible in the MCMC traces. Thus, we discard this simulation from further analysis.

To evaluate the inference, we compute the relative bias (i.e. $\frac{median-true}{true}$), error (i.e. $\left|\frac{median-true}{true}\right|$), and HPD width (i.e. $\frac{upper-lower}{true}$, considering the limits of the 95% HPD intervals) of each estimate. We assess the 95% coverage as the percentage of simulations, in which the true parameter value is in the 95% HPD interval of the estimate. For each parameter, we ascertain that the number of 95% HPD intervals containing the true value falls within the expected range (90 to 99, given 100 simulations) (Mendes et al. 2025).

Second, we simulate phylogenetic trees with varying tree size, i.e. 10, 100, 1000, and 5000 tips. We use a fixed set of parameters, i.e. k=5, ℓ=10, d=0.1, ρ=0.1, and generate 10 trees per size. For the inference, we set prior distributions on the parameters as follows:


$$\begin{array}{r} \begin{array}{cc} k∼\text{LogNormal}(2,1) & \;[1,38]\\ \ell ∼\text{Uniform}(1,100) & \;[3.475,97.525]\\ d∼\text{Beta}(2,5) & \;[0.02,0.59]\\ \rho ∼\text{Beta}(2,5) & \;[0.02,0.59]. \end{array} \end{array}$$

We use the same MCMC settings and summarize the posterior distributions of parameters as before.

### Empirical data analysis

#### Mouse embryonic stem cells

We analyze the intMEMOIR dataset (Chow et al. 2021; Gong et al. 2021) as processed by Seidel and Stadler (2022). In the experiment, the development of 106 cell colonies was traced for 54h using time-lapse microscopy and an integrase-based synthetic barcode system. Crucially, some cells in the colonies did not survive until the end of the experiment, dropped out from the recordings, or the readout of their lineage barcode is missing. Thus, the dataset consists of 106 cell phylogenies with 3–39 tips, comprising a sample of extant cells (25−100%) compared to the recorded cell population trees.

The intMEMOIR recordings have previously been analyzed with the Bayesian phylogenetic framework TiDeTree (Seidel and Stadler 2022) using the constant-rate BD model with sampling, as implemented in BDSKY (Stadler et al. 2013). There, the goal was to reconstruct cell phylogenies from sequence alignments by modeling the editing process. In this analysis, we focus on characterizing cell population dynamics based on phylogenies.

For the purpose of a fair comparison, we re-estimate the cell division rate *λ* and death rate *μ* under the constant-rate BD model with sampling, providing ground-truth phylogenies as input to the inference. We condition on the root (i.e. time of the first cell division) and define priors on the mean cell lifetime $\ell =\frac{1}{\lambda +\mu }$ and death probability $d=\frac{\mu }{\lambda +\mu }$. Next, we estimate the phylodynamic parameters *k*, ℓ and *d* under the age-dependent branching model using our package ADB with analogous settings.

For inference, we set the prior distributions as follows:


$$\begin{array}{r} \begin{array}{cc} k∼\text{LogNormal}(2,1) & \;[1,38]\\ \ell ∼\text{LogNormal}(2.5,1) & \;[0.32,63.19]\\ d∼\text{Beta}(2,5) & \;[0.02,0.59]. \end{array} \end{array}$$

We use the long-tailed log-normal distribution for the shape parameter, considering the low number of stages in the cell cycle, while allowing for higher levels of synchronicity of the cell divisions. The remaining priors are chosen to be broad and only weakly informative, similar to the priors in the previous analysis of this data.

For both phylodynamic models, we run 5 independent MCMC chains with $10^{6}$ iterations for 24h and verify their convergence. We assert that all inferred parameters have ESS >200 and a GR statistic (Gelman and Rubin 1992; Vats and Knudson 2021) near 1 (below 1.01, see Fig. S14). We remove 10% of burn-in and combine the chains to obtain the posterior distribution of parameters.

Independently, we estimate the mean cell lifetime $\hat{\ell }$ and death probability $\hat{d}$ from complete cell population trees. Note that the time scale of the trees is given in movie frames, which were captured approximately every 15 minutes. When converting to hours, the timing of a cell division or death event can only be determined within a 15 minute window around the truth. We derive $\hat{\ell }$ by averaging over the lengths of internal branches and branches ending with a death/ dropout event, and $\hat{d}=\frac{\#death}{\#division+\#death}$ by counting the number of cell divisions and deaths/ dropouts across all trees. We use these empirical measures as reference to assess the accuracy of the phylodynamic parameters inferred from phylogenies.

Next, we simulate 106 phylogenies, each representing the growth of a stem cell colony, under the BD and ADB models. We set the sampling probability per colony to the observed value, and the remaining phylodynamic parameters to the inferred posterior medians. Then, we compute the distribution of internal branch lengths across phylogenies. Additionally, we calculate the B1 index (Shao and Sokal 1990) for each phylogeny, normalizing by the number of tips, to compare the balance of empirical and simulated trees.

#### Arthropod limb

The reconstructed lineage of the *Parhyale* T2 limb (Wolff et al. 2018; Salvador-Martínez et al. 2021) contains multiple tracks, arising from 34 founder cells. The cells were imaged at 400 time-points and tracked over 50 hours of development. For our analysis, we separate the tracks into subtrees, but assume a common underlying tree-generating process. As the lineage relationship of some founder cells is known and part of the process, we obtain 20 subtrees. These trees contain non-extant tips, most likely resulting from cell death, or cell migration towards the inside of the limb, which limited tracking by the software. For phylodynamic inference, we prune the non-extant lineages and consider only subtrees with at least 3 tips. After processing, the dataset contains 17 time-scaled phylogenies with 4–56 extant cells.

Next, we estimate all ADB model parameters from the phylogenies in BEAST2. Again, we use weakly informative prior distributions


$$\begin{array}{r} \begin{array}{cc} k∼\text{LogNormal}(2,1) & \;[1,38]\\ \ell ∼\text{LogNormal}(2.5,1) & \;[0.32,63.19]\\ d∼\text{Beta}(2,5) & \;[0.02,0.59]\\ \rho ∼\text{Beta}(5,2) & \;[0.41,0.98] \end{array} \end{array}$$

and condition on the time of the root. We run 5 independent MCMC chains until convergence, remove 10% of burn-in, and combine the chains for downstream analysis.

## Supplementary Material

msag080_Supplementary_Data

## Data Availability

Code to reproduce the analyses presented in this paper is available at: https://github.com/pilarskj/ADB-analysis. The BEAST2 package is available at: https://github.com/pilarskj/ADB. Our R package for simulating trees under ADB (along with other single-cell simulation tools) is available at: https://github.com/scCEVO/scTreeSim.
