# Supplementary material for: Age-dependent Phylodynamics with Application to Single-cell Lineage Trees
Source: Mol Biol Evol. 2026 Mar 25;43(4):msag080. doi: 10.1093/molbev/msag080 (PMC13049366; doi:10.1093/molbev/msag080)
Supplement: msag080_Supplementary_Data [file msag080_supplementary_data.pdf]

Supplementary Material:
Age-dependent phylodynamics with application to single-cell lineage trees

Nicola Mulberry[*,1,2], Julia Pilarski[*,1,2], Jana Dinger[1], and Tanja Stadler[1,2]

[*]Authors contributed equally
[1]Department of Biosystems Science and Engineering, ETH Zürich, Basel, Switzerland
[2]Swiss Institute of Bioinformatics, Lausanne, Switzerland

A. Tree simulations & lineage-through-time plots

The R package *scTreeSim* is used to generate phylogenies under the ADB process. One way to validate these simulations is by comparison to the expected lineage-through-time plots (dLTT), the equation for which is derived in the main Methods. Overall, we see that the simulator appears to behave as expected (Figure S1). The dLTT curves also give us intuition for our model parameters. We see that, even in low sampling regimes, there is a strong characteristic profile associated to the phylogenies under a high shape parameter, indicative of synchronicity.

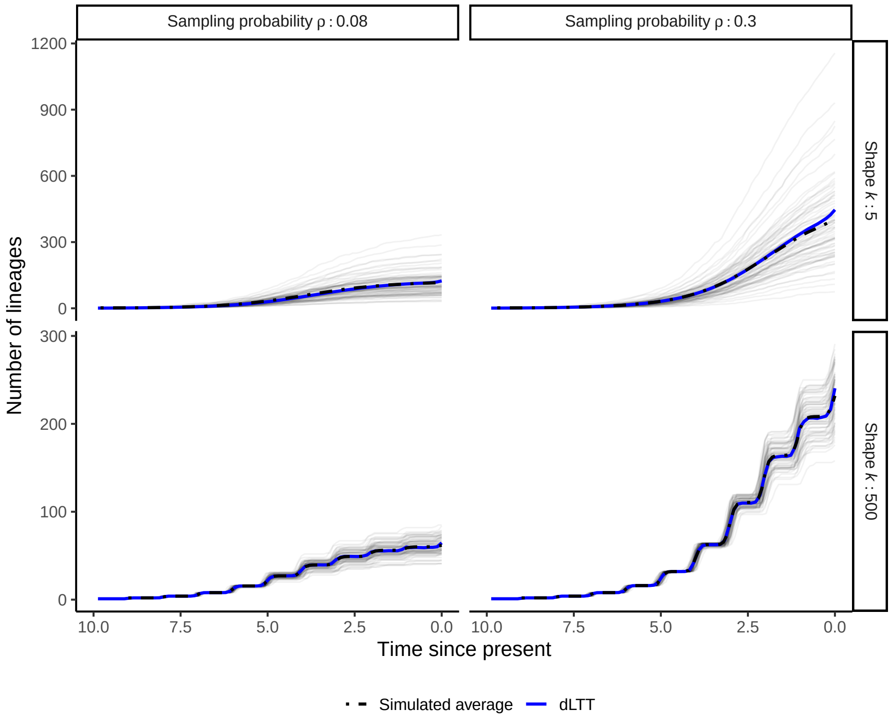

Figure S1: Validation of simulated phylogenies from *scTreeSim* against the expected lineages through time curves (dLTT). Simulations (in grey) are repeated 75 times and the dLTT plot (in blue) is given by Equation 8 in the main text. The average over the simulated curves is shown in black. The death probability is fixed at $d = 0$ for convenience.

B. Approximating edge densities

We now let the cell lifetimes be Erlang distributed. That is, $a \sim \Gamma(k,\theta)$ with integer shape parameter k. We first re-write the quantity $b(s,t)$ in an explicit form: the edge (s,t) represents either a single full life-cycle ending in a branching event at time s, or two life-cycles with branching events at $w_1 \in (s,t)$ and s, and so on. Let $b_n(s,t)$ be the probability that edge (s,t) resulted from n unobserved internal branching events, ending with one at time s. Therefore, we let

$$b(s,t) = \sum_{n=0}^{\infty} b_n(s,t), \tag{1}$$

where

$$\begin{aligned}
b_0(s,t) &= (1-d) f(t-s;k,\theta),\\
b_1(s,t) &= 2(1-d)^2 \int_s^t f(t-w_1;k,\theta) f(w_1-s;k,\theta) P_0(w_1)\, dw_1,\\
&\ \ \vdots\\
b_n(s,t) &= 2^n (1-d)^{n+1} \int_s^t \int_s^{w_1} \cdots \int_s^{w_{n-1}} f(w_n-s) \prod_{i=1}^{n} f(w_{i-1}-w_i) P_0(w_i)\, dw_1 \cdots dw_n.
\end{aligned}$$

In the above equation, we take $w_0 = t$ and f to be the pdf of the Erlang distribution.

We now linearize each term by setting $P_0(\tau) \equiv \overline{P_0}$ for $s \le \tau \le t$. Under the assumption that the lifetimes are Erlang, we can then write

$$\widetilde{b}_n(s,t) = 2^n (1-d)^{n+1} f(t-s;(n+1)k,\theta) \overline{P_0}^{\,n}. \tag{2}$$

We now need to ensure that we take sufficient terms in this approximation. Note that

$$\widetilde{b}_n(s,t) \le 2^n f(t-s;(n+1)k,\theta),$$

and that the maximum of the equation above occurs around $n^* = \lfloor 2^{1/k}(t-s)/(k\theta) \rfloor$ (in reality, the maximizer of $\widetilde{b}_n(s,t)$ is often lower). Therefore, for some user-specified tolerance $\epsilon << 1$, our approximation thus becomes

$$b(s,t) \approx \sum_{n=0}^{n_u} \widetilde{b}_n(s,t), \tag{3}$$

where $n_u \ge n^*$ such that $\widetilde{b}_n(s,t) \le \epsilon$.

C. Accuracy of Likelihood Computation

We first test our implementation of the ADB model in BEAST2 by comparing the likelihood calculation for $k = 1$ to the analytical solution under the constant-rate BD process as implemented in the package BDMM-Prime [1]. We see a good agreement of the likelihood curves with both the "exact" and approximated edge densities (Fig. S2).

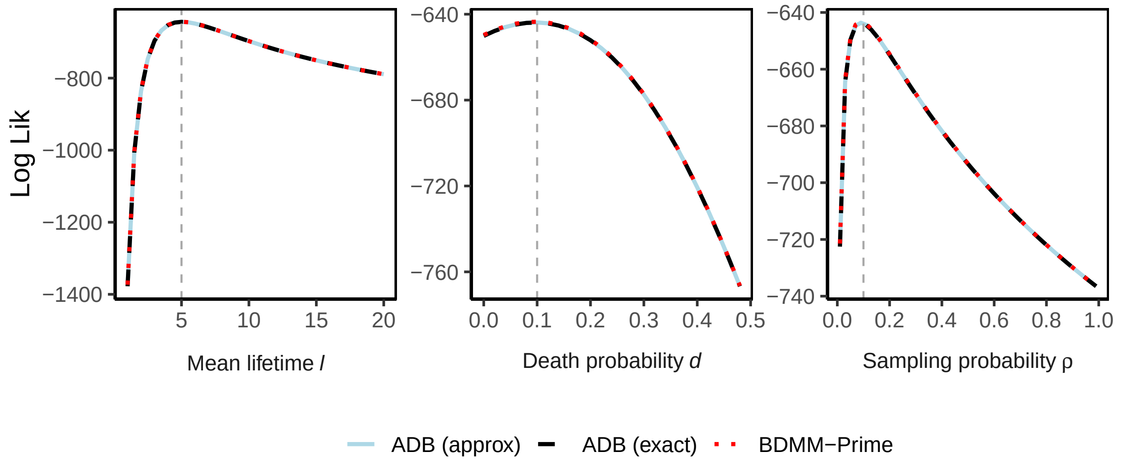

Figure S2: Match of likelihood curves calculated with ADB and BDMM-Prime for $k = 1$. The graphs show the log-likelihood (y-axis) for different parameter values (x-axis) for a tree with 100 tips, $t_{or} = 50$, and true parameters $\ell = 5, d = 0.1, \rho = 0.1$ (indicated by dashed lines) simulated with TreeSim [2]. The ADB parameters correspond to birth rate $\lambda = 0.18$ and death rate $\mu = 0.02$ in the constant-rate BD process.

However, we observe that in case of very low sampling, i.e. $\rho \lesssim 0.01$, the ADB likelihood deviates from the analytical solution at default settings (i.e., using a step size of 2^{14} for FFT when solving the P_0 and P_1 integral equations). The numerical errors decrease at higher step size for FFT in P_0 and P_1 calculations (Fig. S3). For larger k, the errors are further reduced by forcing P_0 to be non-increasing if $1 - \rho > d/(1 - d)$, and non-decreasing otherwise. We find that the low ρ and high d parameter regimes are particularly difficult to resolve, and thus, the numerical solution requires taking more sample points than the default setting.

Conversely, we find that in higher sampling regimes, i.e. $\rho \gtrsim 0.1$, the ADB likelihood is accurate already at a lower than default step size for FFT. On Figure S4A, we present a more systematic evaluation of the likelihood error under ADB at varying sampling probability ρ. Note that reducing the step size increases the accuracy, but substantially reduces the runtime per likelihood calculation (Figure S4B).

A

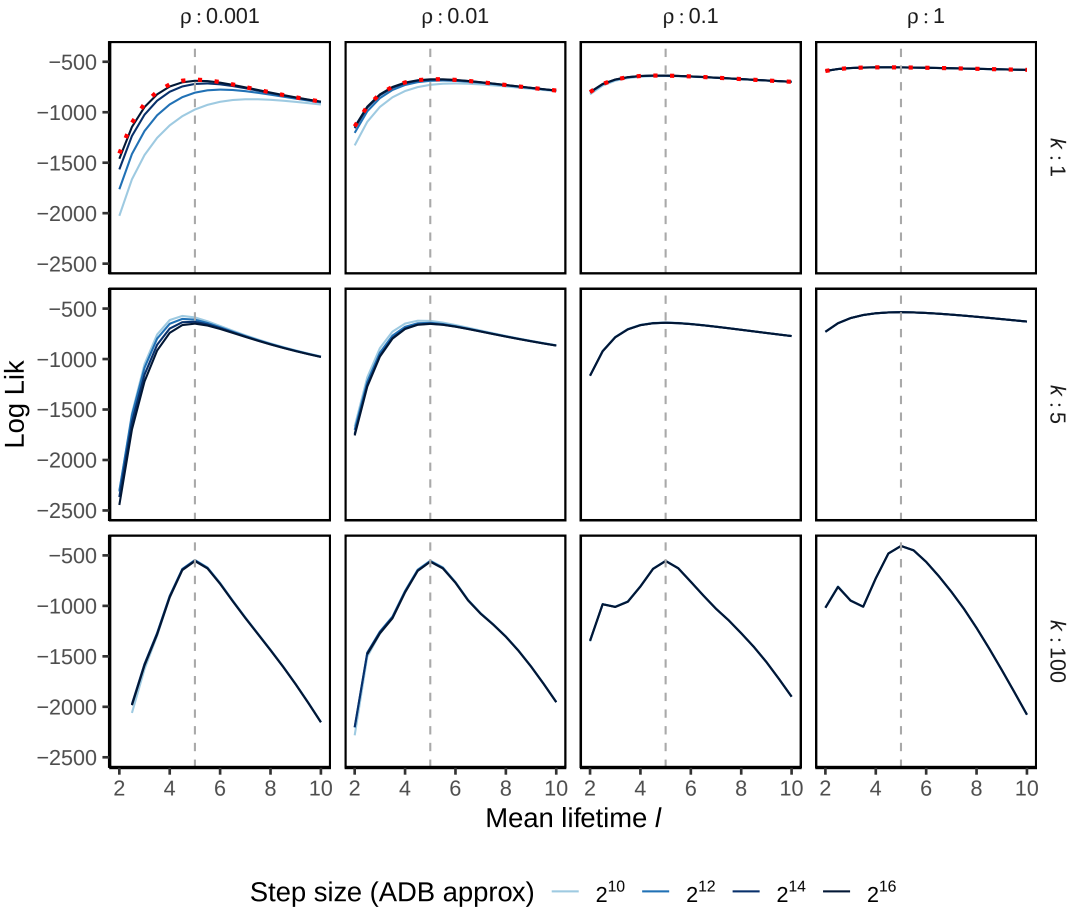

Figure S3: Likelihood curves at varying shape k and sampling probability ρ. For each panel, we simulated one tree with 100 tips and parameters $\ell = 5, d = 0.1$ (as indicated by dashed lines), and k, ρ (as indicated by row and column labels). We calculated the likelihood under ADB (with approximation) for a range of ℓ (**A**) and d (**B**) parameter values, fixing the remaining parameters to the values used for simulation. The error decreases with increasing FFT step size (line colors) when solving the integral equations for P_0 and P_1. For $k = 1$, we compare against the analytical BD solution (red dotted line).

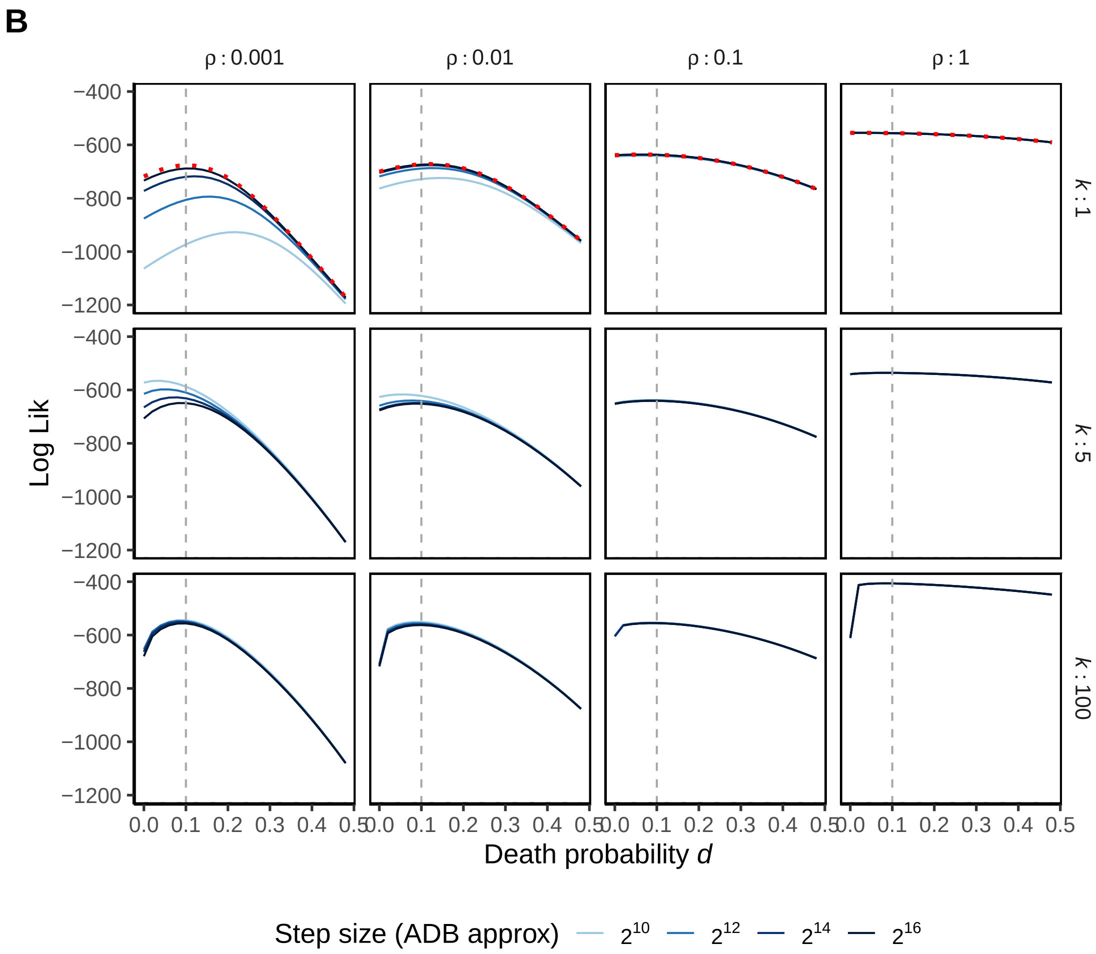

Figure S3: Likelihood curves at varying shape k and sampling probability ρ. (cont.)

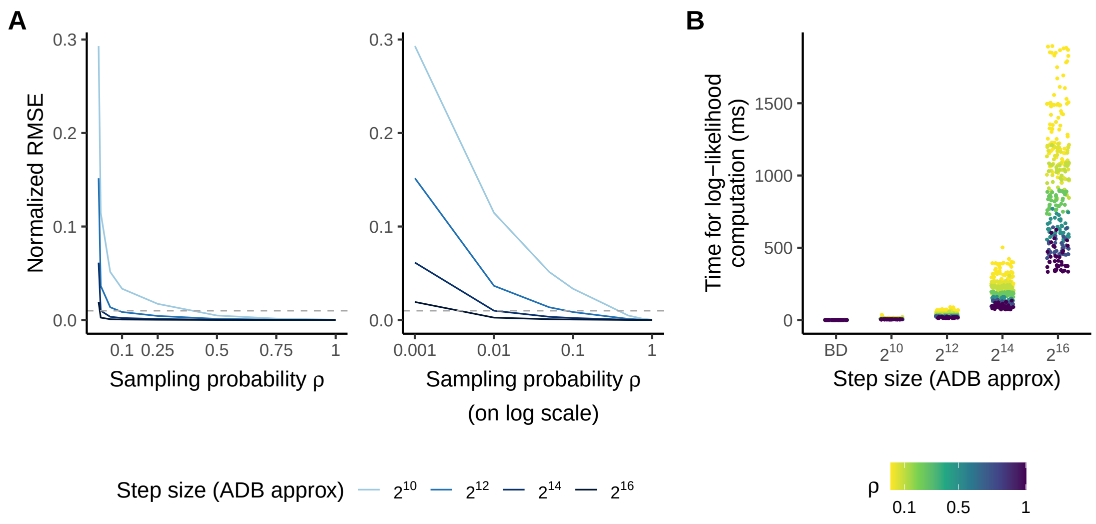

Figure S4: Likelihood calculation at varying sampling probability ρ. **A**: Error. For a range of ρ values (x-axis), we simulated one tree with 100 tips and parameters $k = 1, \ell = 5, d = 0.1$ with *scTreeSim*. For each tree, we calculated the likelihood under ADB (with approximation) for a range of ℓ parameter values (from 2 to 20), fixing the remaining parameters to the truth. We used different step sizes for FFT in P_0 and P_1 calculations (line colors). We evaluated the root mean square error (RMSE, y-axis) of the log-likelihood values wrt. the BD analytical solution, normalized over the reference range. Setting an error threshold of 0.01 (dashed grey line), we observe sufficient accuracy of the likelihood calculations with a step size of at least 2^{14} for $\rho \approx 0.01$ and 2^{12} for $\rho \gtrsim 0.1$. **B**: Runtime per likelihood calculation.

Next, we investigate the error in the likelihood calculation due to approximating edge densities (cf. Section 4.2). For each edge (s,t), the error is a combination of the truncation error – which we can control by taking a sufficient number of terms $\tilde{b}_n(s,t)$ to obtain $\tilde{b}(s,t)$ – and an intrinsic error per term $\tilde{b}_n(s,t)$ arising when P_0 is not constant along the edge. In this case, $\tilde{b}(s,t)$ might not converge to the exact solution $b(s,t)$. In practice, the intrinsic error results in decreasing accuracy of the likelihood calculation for small d, as shown in Figure S5.

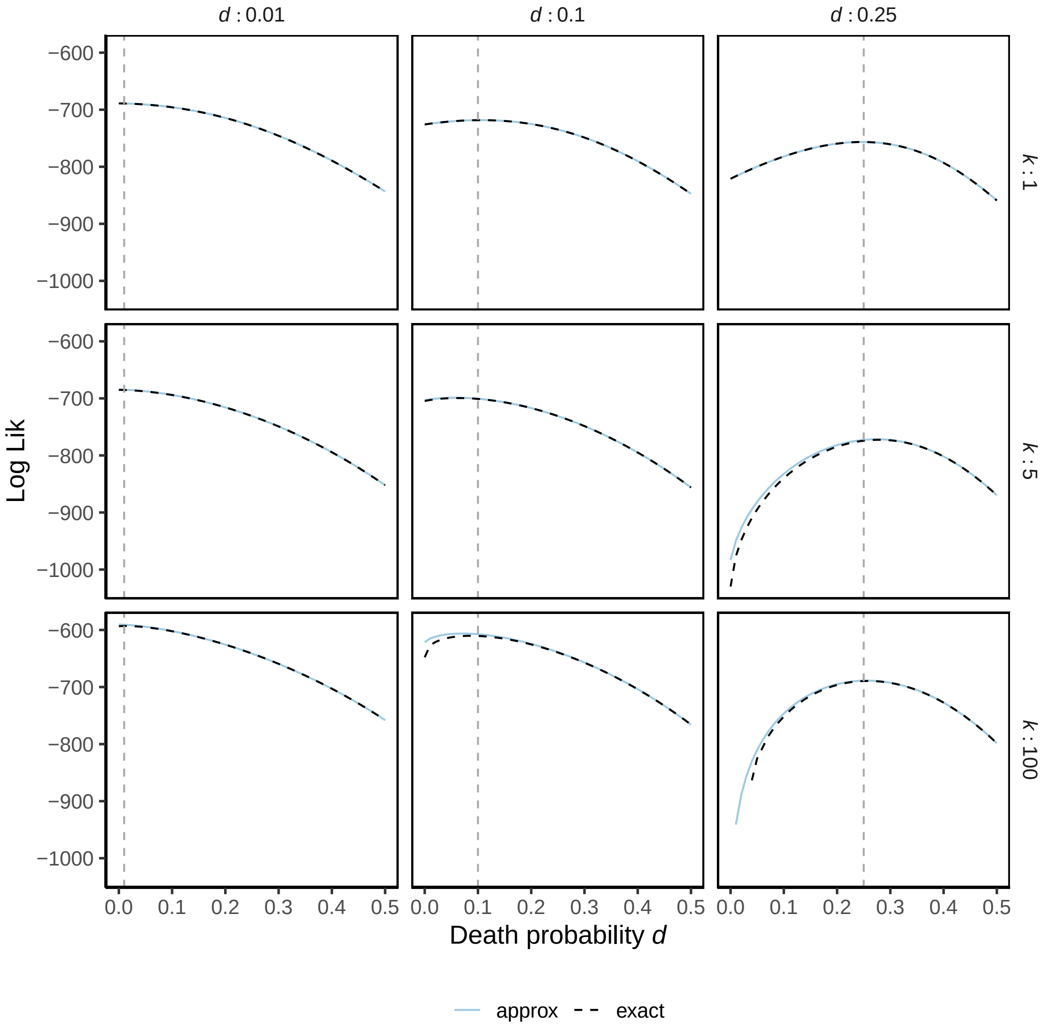

Figure S5: Likelihood curves for trees with varying shape parameter k (rows) and death probability d (columns and dashed vertical lines), $\ell = 10$, $\rho = 0.1$, and 100 tips simulated with *scTreeSim*. Each curve was obtained by computing the log-likelihood under ADB (with or without approximation) for a range of d parameter values, fixing the remaining parameters to the truth. The approximation loses precision for small d.

D. Accuracy of Bayesian Phylodynamic Inference

As described in Section 5.6, we simulated 100 phylogenetic trees with 100 tips under the ADB model with parameters drawn from their prior distributions, and re-estimated the parameters using Bayesian phylodynamic inference. Here, we perform coverage validation [3] of the ADB model by calculating the $100 \times \alpha\%$ HPD interval for each tree and parameter, considering a range of credibility levels $\alpha \in (0, 1)$. In Figure S6, we report the percentage of simulations containing the correct data-generating parameter in $100 \times \alpha\%$ HPD interval. We attribute minor deviations from the expected bounds to the relatively low number of simulations, broad priors, and the two sources of errors described before: numerical errors at solving integral equations for P_0 and P_1, and errors due to approximating the edge densities.

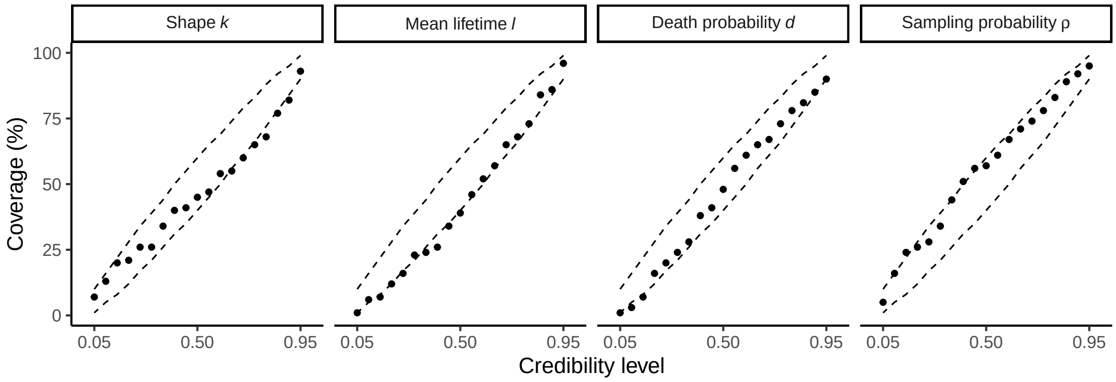

Figure S6: Coverage validation. Dots represent the percentage of simulations (y-axis) containing the true parameter in $100 \times \alpha\%$ HPD interval for a range of credibility levels α (x-axis). Dashed ellipses indicate expected bounds.

To further detect and characterize biases in parameter estimates, we perform rank-uniformity validation (RUV), as described in Mendes et al. [3]. As shown on S7, the ranks for each parameter are compatible with a uniform distribution within the confidence region.

Additionally, in Figure S8, we plot the relative bias, error and 95% HPD interval width of the inferred parameters with respect to true parameter values per simulation.

In our simulation study, we have further observed a systematic bias in the inference of death probabilities on large trees. In Figure S9, we plot the likelihood curves with respect to d for three example trees with 5000 tips. In line with our investigation in Section C, we observe an increasing approximation error for small d. This error results in a shift of peaks of the likelihood curves to smaller values. The inferred parameters align with this shift. Note that despite the approximation error, the true parameter $d = 0.1$ has been recovered in 8 out of 10 simulations.

To assess the impact of the bias on inference in various parameter regimes, we repeated the simulations and validation on tenfold larger trees, that is, with 1000

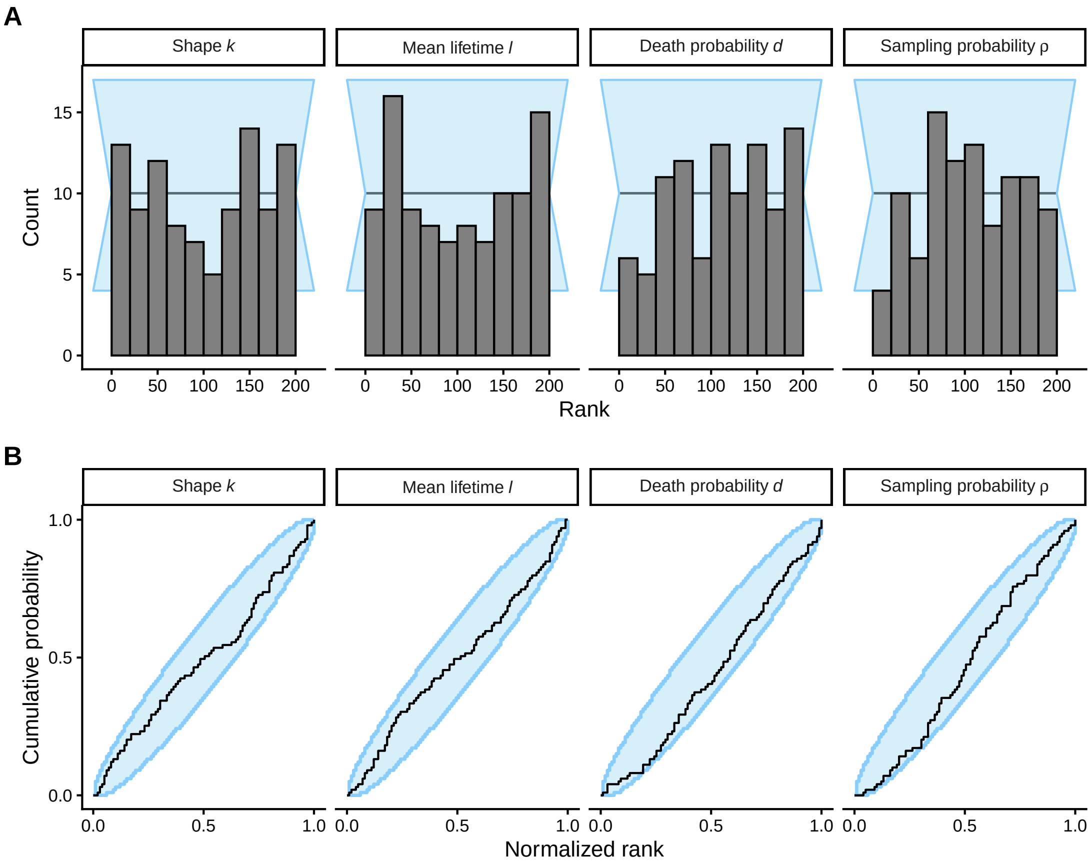

Figure S7: RUV validation. **A**: Rank histograms. For each parameter, the plot shows the distribution of ranks of parameter values (sampled from the prior) relative to their corresponding posteriors (obtained after 10% burn-in and thinning down to 200 MCMC samples). **B**: Corresponding empirical cumulative distribution function (ECDF) plots. Light-blue bands and ellipses represent 95% confidence intervals about the expected rank count and ECDF, respectively.

tips (Figure S10). Indeed, the coverage of death probability d dropped to 78%. The rank histogram of d is skewed to the right which agrees with the observed underestimation of this parameter. Importantly, the bias does not seem to hinder reliable inference of the remaining parameters, which pass the 95% coverage criterion. The HPD intervals around the estimates are narrower in case of larger trees, as expected with the increase in data. Overall, all inferred parameters, including d, exhibit strong correlation between the posterior medians and the true values.

In Section C, we described numerical errors in the likelihood computation at low sampling probability ρ (in particular, for low k) and the approximation error at small d. Here, we perform simulations in these problematic parameter regimes to assess the practical limitations of the ADB package for phylodynamic inference. We simulate 20 trees with 100 tips under ADB with parameters drawn from prior

distributions

$$\begin{aligned} k &\sim \text{Uniform}(1, 100) && [3, 98] \\ \ell &\sim \text{LogNormal}(2, 1) && [0.19, 38.33] \\ d &\sim \text{Exponential}(20) && [3.17 \times 10^{-10}, 0.15] \\ \rho &\sim \text{Beta}(2, 200) && [2.16 \times 10^{-4}, 2.35 \times 10^{-2}] \end{aligned}$$

For each tree, we run MCMC to infer k, ℓ, d and ρ. For three trees with the lowest shape parameters ($k < 15$), the error in likelihood calculation exploded within a few thousands iterations, pushing k, d and ρ towards the lowest possible values. For the remaining trees (with true $k > 20$), the chains converged. As shown in Figure S11, the parameters k, ℓ and ρ could be inferred accurately, even at a sampling probability of ≈ 0.001. As before, death probabilities d tend to be underestimated. Taken together, we advice to use the ADB package with caution when $k \lesssim 20, \rho \lesssim 0.01$ and take into account potential underestimation of d.

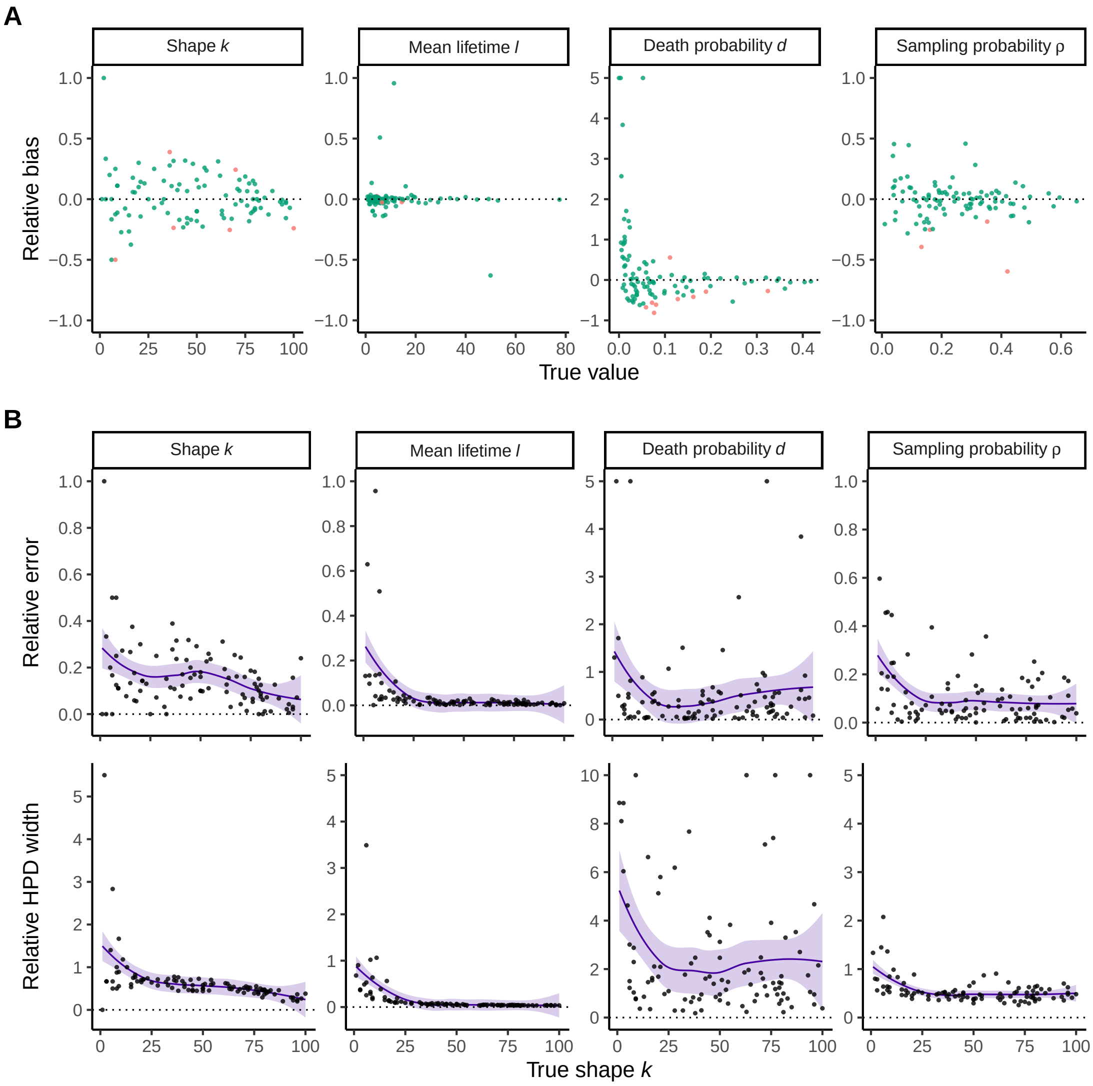

Figure S8: Accuracy of Bayesian phylodynamic inference on simulations. **A**: Relative bias (y-axis) of inferred parameters with respect to true parameter values (x-axis), colored by 95% HPD coverage. **B**: Relative error (top) and HPD width (bottom) of inferred parameters with respect to true shape parameter k. Purple lines represent trend lines with 95% confidence bands. For readability, large values arising through division by near-zero death probabilities are clipped.

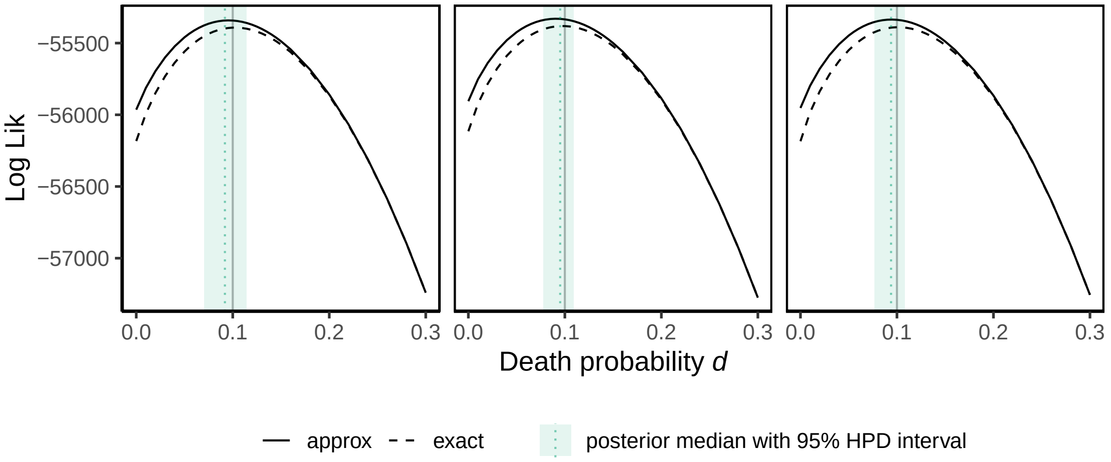

Figure S9: Bias in inferring death probabilities due to approximation error at low d. Shown are likelihood curves for three trees with 5000 tips selected from the simulation study (with true parameters $k = 5, \ell = 10, d = 0.1$ and $\rho = 0.1$). Solid vertical lines indicate the true parameter, dashed lines with ribbon indicate the inferred parameters.

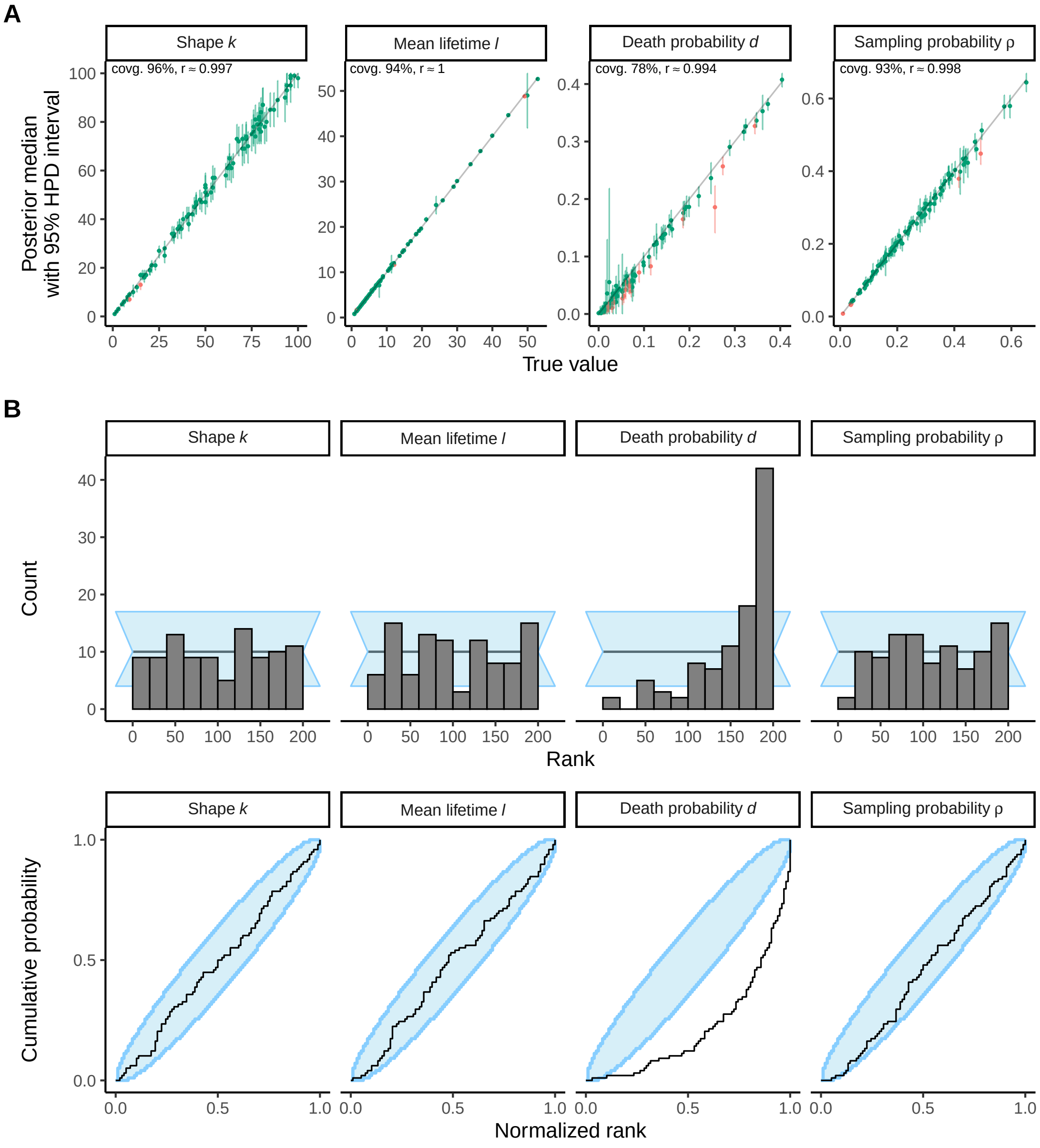

Figure S10: Validation of ADB model on simulated trees with 1000 tips. **A**: Coverage. Panels show the true parameter values (x-axis) plotted against the posterior estimates (y-axis) for 98 out of 100 simulations for which the MCMC reached convergence. Dots indicate the medians, bars the 95% HPD intervals, and the diagonal line shows $x = y$. Simulations for which the 95% HPD intervals contain the true value are highlighted in green, otherwise in red. The percentage summarizes the coverage, r denotes the Pearson correlation coefficient between the posterior medians and true parameter values. **B**: RUV. Rank histograms (top) and corresponding ECDF plots (bottom).

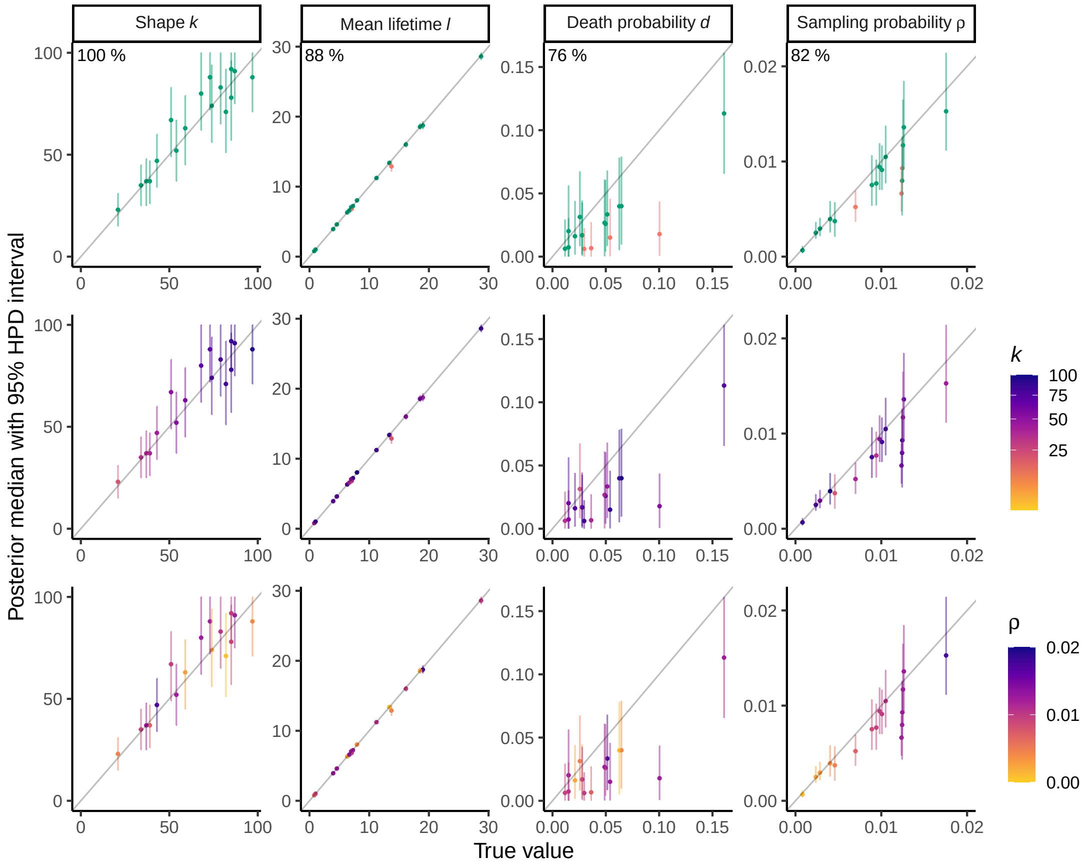

Figure S11: Phylodynamic inference in erroneous parameter regimes. Panels show the true parameter values (x-axis) plotted against the posterior estimates (y-axis) for 17 out of 20 simulations for which the MCMC reached convergence. Dots indicate the medians, bars the 95% HPD intervals, and the diagonal line shows $x = y$. In the top row, simulations for which the 95% HPD intervals contain the true value are highlighted in green, otherwise in red. The percentage summarizes the coverage. In the middle row, simulations are colored by shape k. In the bottom row, simulations are colored by sampling probability ρ.

E. Identifiability

We aim to understand how the known non-identifiability of the constant-rate birth-death-sampling model [4] propagates with higher shape parameters. In particular, assuming the ADB model is the true generative model, we investigate whether all parameters can be estimated. Due to the analytical intractability of our likelihood, we explore here the question of *practical* non-identifiability through simulations and numerical studies.

In our simulation study, we were generally able to infer all four population-dynamic parameters, however we found that parameters inferred from trees with smaller shape parameter k exhibit a larger bias and uncertainty. Specifically, the trend lines in Figure S8 indicate an increase in relative 95% HPD widths for smaller k. In this regime, we observe potential parameter non-identifiability, as visible also in Figure 2. When $k \lesssim 20$, while the shape parameters themselves are well-inferred, there is significant uncertainty in the inference of the remaining three parameters. We seek to explore this non-identifiability further.

For the case of shape parameter $k = 1$, we recall the analytical expressions for non-identifiable parameter transformations $(k = 1, \rho, l, d)$ and $(k = 1, \rho', l', d')$ generate equal processes if and only if:

$$l' = \frac{l}{d - (1-d)(1-2\rho/\rho')} \quad \text{and} \quad d' = \frac{d - (1-d)(1-\rho/\rho')}{d - (1-d)(1-2\rho/\rho')}. \tag{4}$$

For $k > 1$, we lack an analytical likelihood, and so we investigate possible non-identifiabilities using the deterministic lineage-through-time curves (dLTTs), since any two models with the same dLTT yield identical likelihoods for a given tree [5]. No closed-form solution exists for parameters generating equal dLTTs, but we can draw on numerical methods to generate and compare curves.

We first ask, for a fixed k, can we find (practically) congruent processes, one with high sampling and one with low sampling? Figure S12 shows an example where, starting with the known non-identifiability at $k = 1$, we can find qualitatively similar dLTTs in each regime. Finding suitable parameters becomes increasingly difficult as k increases.

In most of our simulation studies, we have found the shape parameter k to be strongly identifiable. An exceptional case occurs when d is very high ($d \gtrsim 0.45$). For large values of k, the dLTT approaches a piecewise-constant function with step-size l. As a result, different lifetimes can no longer produce identical dLTTs, making theoretical non-identifiability impossible. However, practically speaking, when d is large, we can find parameter sets where a process with a high shape parameter closely approximates one with a lower shape parameter (Figure S13).

All of our simulations thus far have indicated that we can reliably infer all four parameters (k, d, ℓ, ρ) as long as we ensure that d is not too large and that we are sufficiently far away from a "birth-death-like" process (i.e. $k >> 1$). In practice, we suggest putting semi-informative priors on the death probability d, and to fix the sampling proportion ρ unless there is strong prior information that $k \gtrsim 20$.

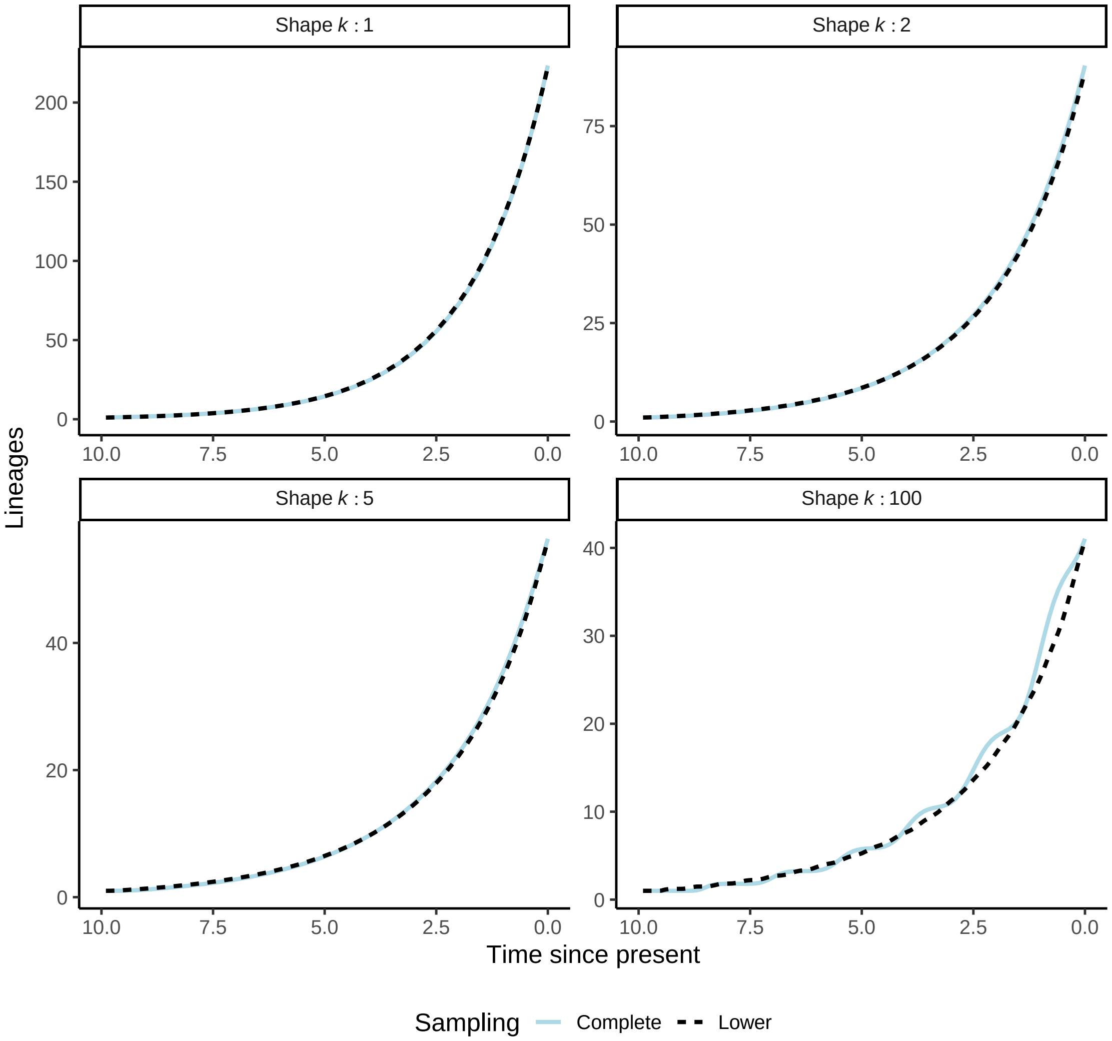

Figure S12: Examples of similar dLTTs. For the fully sampled case, we take parameters $(k, \rho = 1, \ell = 1.5, d = 0.1)$. For each given shape parameter, we set $(k' = k, \rho' = 0.5, \ell' = 0.57, d' = d_k)$, where d_k is the best match found through a grid search.

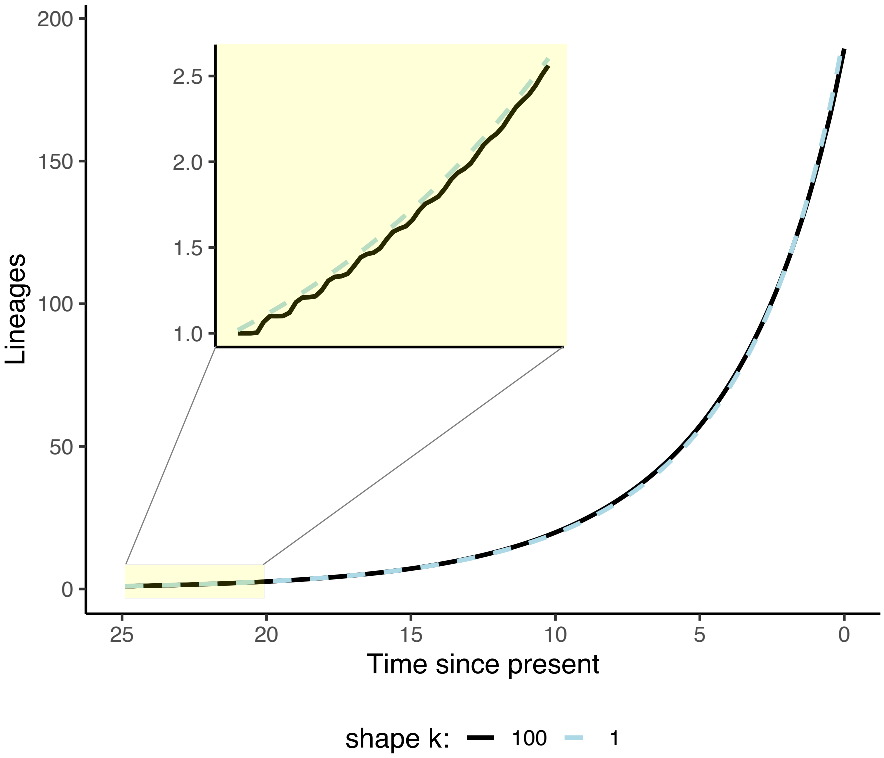

Figure S13: Scenario where a high shape parameter appears to approximate the dLTT with a lower shape parameter. For $k = 100$, we take $d = 0.45$.

F. intMEMOIR Analysis

Below, we report the Gelman-Rubin convergence diagnostics [6, 7] for the empirical data analysis described in Sections 3.2 and 5.7. Considering the 5 independent MCMC runs under ADB, we obtain

$$\hat{R}_k = 1.000026$$
$$\hat{R}_\ell = 1.000042$$
$$\hat{R}_d = 1.001810$$

Figure S14 shows the evolution of the shrink factor as the number of MCMC samples increases, which stabilizes near 1 for all parameters.

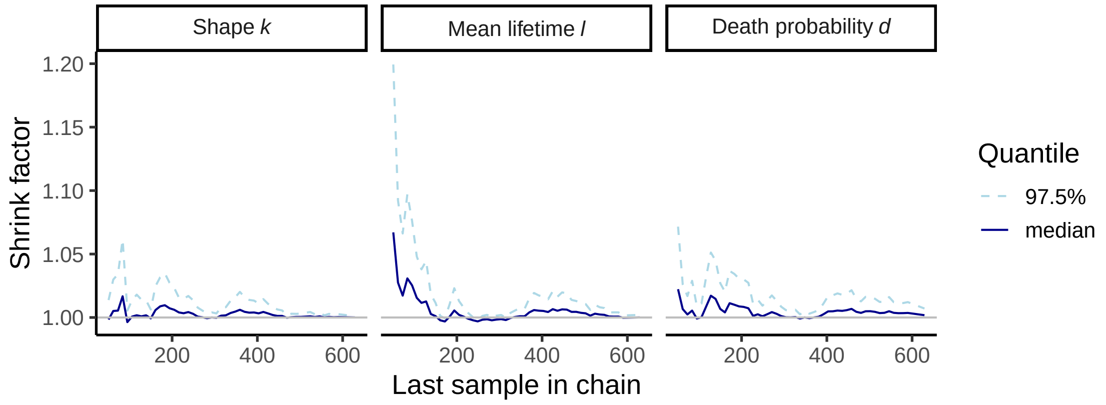

Figure S14: Convergence of the phylodynamic inference from intMEMOIR cell phylogenies. The panels show the evolution of Gelman and Rubin's shrink factor per ADB parameter as the number of MCMC samples increases.

As mentioned in Section 5.7, the intMEMOIR dataset contains cell population trees from time-lapse microscopy movies, but also genetic lineage barcodes for a sample of cells per colony [8]. For each tip in a cell phylogeny, i.e., sampled cell at final time point, an array of length 10 indicates the state of the recording DNA sequence at site $i = 1, ..., 10$. Each sequence could either be unedited, or acquire an inversion or deletion – the edits across sites accumulated over time.

Seidel and Stadler [9] have previously analyzed the intMEMOIR recordings with the Bayesian phylogenetic framework TiDeTree. They jointly inferred cell phylogenies and parameters of the editing and phylodynamic models from sequence alignments. Hence, the editing model settings affected phylodynamic estimates. When fixing phylogenies in the inference, the editing and phylodynamic parameters become independent. In this case, the posterior distribution over the space of parameters follows

$$\mathbb{P}(\Phi, \Psi | D, \mathcal{T}) \propto \mathbb{P}(\Phi)\mathbb{P}(\Psi)\mathbb{P}(\mathcal{T}|\Psi)\mathbb{P}(D|\mathcal{T}, \Phi) \tag{5}$$

where Ψ denotes the phylodynamic model parameters and Φ represents the editing model parameters.

As a consequence, we do not expect the editing model parameters to differ when using different phylodynamic models. Indeed, when jointly inferring editing model parameters with either the BD or ADB model parameters, we obtain consistent estimates, as shown in Figure S15. Furthermore, the estimates agree with the previous analysis of the data, in which the editing rates r_i and the edit-outcome rate multipliers (i.e. for acquiring an inversion s_i^I, or a deletion s_i^D) are allowed to vary across sites and the following prior distributions are used:

$$r_i \sim \text{LogNormal}(-5, 1) \qquad [0.0002, 0.035]$$
$$s_i^I, s_i^D \sim \text{LogNormal}(0, 1) \qquad [0.03, 5.17].$$

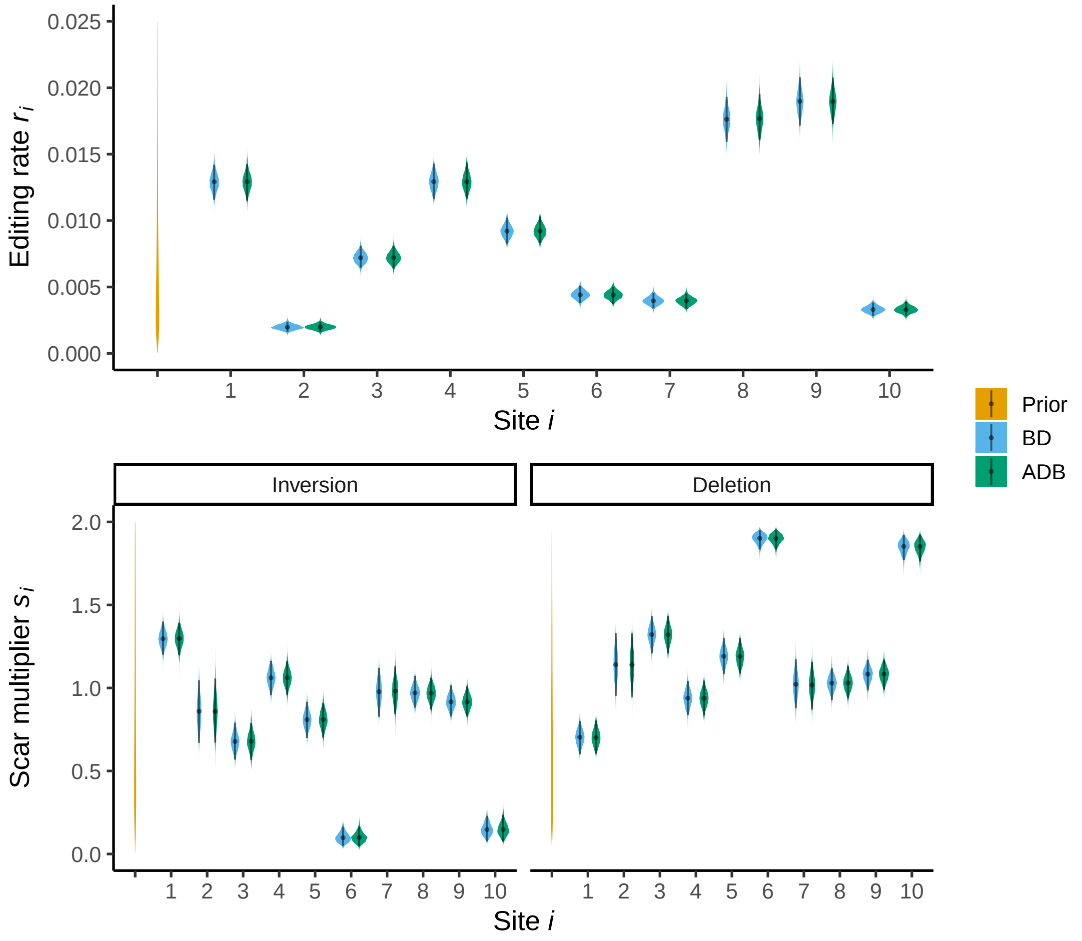

Figure S15: Inference of editing dynamics in intMEMOIR recordings. The graphs show prior and posterior distributions of editing model parameters under TiDeTree, jointly inferred with phylodynamic parameters under the BD and ADB models.

In a supplementary analysis, we run phylodynamic inference on the intMEMOIR dataset with estimating all ADB model parameters, including the sampling probability ρ. That is, we infer the shape parameter k, mean lifetime ℓ, and death

probability d jointly from all cell phylogenies, and additionally, a sampling probability ρ for each of the 106 trees. We use a broad prior, $\rho \sim \text{Uniform}(0,1)[0.025, 0.975]$. Despite small tree sizes, and thus noisy input data, we recover the sampling proportion for 84 out of 106 trees. For these trees, the observed proportion is in the 95% HPD interval of the estimate (if $\rho < 1$, otherwise we verify that the posterior distribution approaches 1, specifically, the upper bound of the interval ≥ 0.999). The mean of the inferred sampling probabilities (≈ 0.82) roughly agrees with the mean sampling proportion across trees (≈ 0.79). Importantly, the population-dynamic parameters of interest, k, ℓ and d, could be inferred equally well, when the information on sampling was missing (Figure S16).

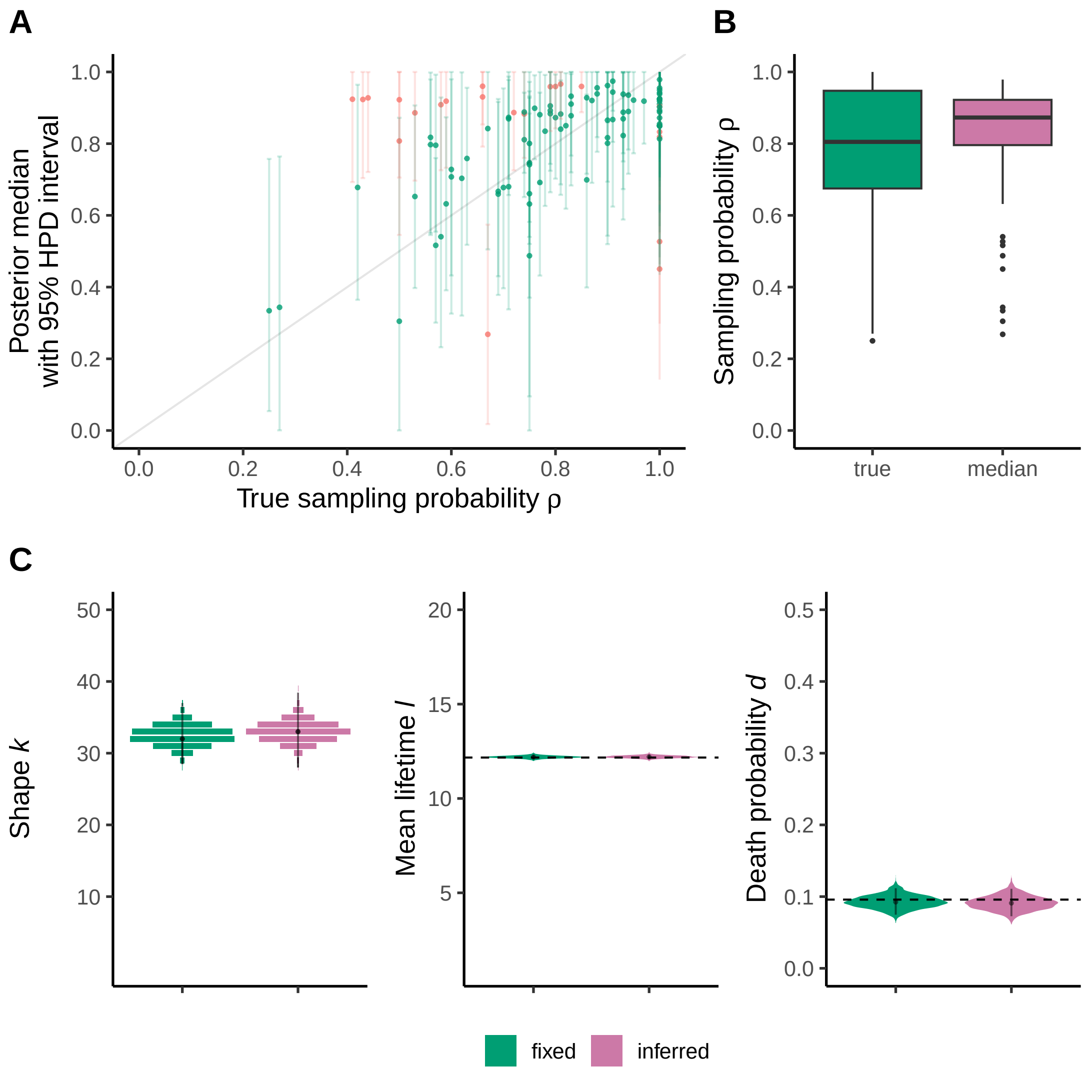

Figure S16: Phylodynamic inference from intMEMOIR cell phylogenies. **A**: Observed sampling proportions (x-axis) plotted against the posterior estimates (y-axis) per tree. Dots indicate the medians, bars the 95% HPD intervals, and the diagonal line shows $x = y$. Recovered proportions are colored in green, otherwise in red. **B**: Distribution of observed (true) and inferred (median) sampling proportions. **C**: Posterior distributions of phylodynamic parameters inferred from cell phylogenies under the ADB model with fixed or inferred sampling probability ρ. Dashed lines indicate the mean lifetime $\hat{\ell}$ and death probability $\hat{d}$ estimated from cell population trees for reference.

References

[1] Timothy G Vaughan and Tanja Stadler. Bayesian phylodynamic inference of multitype population trajectories using genomic data. *Molecular Biology and Evolution*, 42(6):msaf130, 2025.

[2] Tanja Stadler. Simulating trees with a fixed number of extant species. *Systematic Biology*, 60(5):676–684, 2011.

[3] Fábio K Mendes, Remco Bouckaert, Luiz M Carvalho, and Alexei J Drummond. How to Validate a Bayesian Evolutionary Model. *Systematic Biology*, 74(1):158–175, 2025.

[4] Tanja Stadler. On incomplete sampling under birth–death models and connections to the sampling-based coalescent. *Journal of Theoretical Biology*, 261(1): 58–66, 2009.

[5] Stilianos Louca and Matthew W Pennell. Extant timetrees are consistent with a myriad of diversification histories. *Nature*, 580(7804):502–505, 2020.

[6] Andrew Gelman and Donald B Rubin. Inference from iterative simulation using multiple sequences. *Statistical Science*, 7(4):457–472, 1992.

[7] Stephen P Brooks and Andrew Gelman. General methods for monitoring convergence of iterative simulations. *Journal of Computational and Graphical Statistics*, 7(4):434–455, 1998.

[8] Ke-Huan K Chow, Mark W Budde, Alejandro A Granados, Maria Cabrera, Shinae Yoon, Soomin Cho, Ting-Hao Huang, Noushin Koulena, Kirsten L Frieda, Long Cai, et al. Imaging cell lineage with a synthetic digital recording system. *Science*, 372(6538):eabb3099, 2021.

[9] Sophie Seidel and Tanja Stadler. TiDeTree: a Bayesian phylogenetic framework to estimate single-cell trees and population dynamic parameters from genetic lineage tracing data. *Proceedings of the Royal Society B*, 289(1986):20221844, 2022.
